# 2D Metal‐Organic Frameworks for High‐Performance Solid‐State Electrolytes: A Comprehensive Review

**DOI:** 10.1002/advs.202522230

**Published:** 2026-03-02

**Authors:** Changchun Ai, Yuan Tian, Yilei Shu, Peng Hang, Wentao Li, Yiling Yao, Huijuan Guo, Qun Yi, Hongmei Dai

**Affiliations:** ^1^ School of Chemical Engineering and Pharmacy State Key Laboratory of Green and Efficient Development of Phosphorus Resources Wuhan Institute of Technology Wuhan P. R. China

**Keywords:** 2D MOFs, conduction mechanisms, performance modulation, solid‐state electrolytes, structural characterization

## Abstract

Solid‐state electrolytes (SSEs) represent a pivotal pathway for resolving critical battery safety concerns, offering excellent lithium‐ion conductivity, superior chemical compatibility, wide electrochemical stability windows, robust thermal stability, and potential for low‐cost mass production. However, conventional 3D metal‐organic framework (3D MOF)‐based SSEs are often plagued by tortuous ion conduction pathways, poor interfacial contact, and inadequate mechanical properties. In contrast, 2D metal‐organic frameworks (2D MOFs) demonstrate immense potential in the SSE field, owing to their unique layered structures, abundant active sites, and tunable ion‐transport channels. This article systematically reviews the latest research progress in 2D MOF‐based SSEs, with a focus on their structural characteristics, ion transport mechanisms, and interface optimization strategies. We analyze current challenges and offer perspectives on future development directions, providing new insights for the design of high‐performance solid‐state battery electrolytes.

## Graphical abstract

In this review, we elucidate the intrinsic advantages, synthetic methodologies, structural characteristics, ion‐transport mechanisms and performance optimization strategies while highlighting representative material systems and applications of 2D MOFs for electrolyte applications. Specifically, we discuss current challenges and outline future research directions for 2D MOF‐based SSEs.

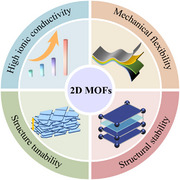

## 1 Introduction

Lithium‐ion batteries (LIBs) are a cornerstone of modern energy storage, yet their safety, environmental impact, and resource sustainability remain key challenges [1, 2, 3]. Conventional LIBs predominantly employ organic liquid electrolytes, which suffer from drawbacks such as poor thermal stability, leakage potential, and flammability, posing significant safety risks and limiting their application in next‐generation, high‐safety, long‐life energy storage systems [4, 5, 6]. To address these issues, SSEs have garnered substantial attention in both academic and industrial sectors. Compared to traditional liquid electrolytes, SSEs offer distinct advantages: (1) Enhanced safety: The absence of flammable organic solvents in SSEs fundamentally mitigates fire and explosion risks [7, 8, 9, 10]; Meanwhile, SSEs have high mechanical strength and can effectively inhibit the growth of lithium dendrites. When lithium dendrites grow in liquid electrolyte, they may puncture the diaphragm and lead to battery short circuit. And the presence of SSEs can act as a physical barrier to the growth of lithium dendrites. This property is also widely recognized as a key factor to enhance battery safety [11, 12, 13]. (2) Dendrite suppression: SSEs possess high mechanical strength, acting as a physical barrier to inhibit lithium dendrite growth—a primary cause of internal short circuits—thereby enhancing battery safety [14, 15]; They also offer a broader operating temperature range due to superior thermal stability and maintained ionic conductivity at low temperatures [16, 17, 18, 19, 20]; (3) Extended lifespan: SSEs can suppress the dissolution of electrode materials, preserving active material integrity and prolonging battery life [21].

Current SSE research focuses on five primary material systems: (1) Solid polymer electrolytes (SPEs): e.g., Poly (ethylene oxide) (PEO), Polyvinylidene Fluoride (PVDF). They offer flexibility and good interfacial contact but suffer from low room‐temperature ionic conductivity (<10^−4^ S cm^−1^) and insufficient electrochemical stability [22, 23, 24]. Our groups have presented a systematic review of PEO‐based SSEs, offering a critical analysis of their advantages and drawbacks alongside a discussion of potential solutions to overcome these challenges [25]; (2) Oxide solid‐state electrolytes (OSEs): They exhibit high chemical/electrochemical stability, mechanical strength, and thermal stability, but being troubled by lower ionic conductivity at room temperature and rigid interface contact problems leading to high impedance at the electrode/electrolyte interface [26, 27, 28, 29, 30], Key systems include: Perovskite‐type (e.g., Li_3x_La_2/3‐x_TiO_3_ (LLTO)): Conductivity ∼10^−4^–10^−3^ S cm^−1^, but Ti^4+^ is easily reduced [31, 32]. Na Super‐ionic Conductor (NASICON) (e.g., Li_1.5_Al_0.5_Ge_1.5_(PO_4_)_3_ (LAGP), Li_1.4_Al_0.4_Ti_1.6_(PO_4_)_3_ (LATP)): Conductivity ∼10^−4^–10^−3^ S cm^−1^, but sensitive to moisture and reactive with Li [33, 34], Garnet‐type (e.g., LLZO): Cubic phase conductivity up to 10^−3^ S cm^−1^, stable against Li, but requires high sintering temperatures [35, 36]; (3) Sulfide Solid‐State Electrolytes (SSEs): e.g., Li_10_GeP_2_S_12_ (LGPS), Li_6_PS_5_Cl (LPSCl). They offer high ionic conductivity (∼10^−2^ S cm^−1^, nearing liquid electrolytes) and good ductility, but are hygroscopic and require interfacial compatibility optimization [37, 38, 39, 40]. (4) Halide Solid‐State Electrolytes (HSEs): e.g., Li_3_YbCl_6_, Li_3_InCl_6_. They provide a balance between OSEs and SSEs, with high conductivity (∼10^−3^ S cm^−1^) and stability against high‐voltage cathodes (>4 V), though compatibility with Li metal anodes can be an issue [41, 42, 43]; and (5) Composite Solid‐State Electrolytes (CSEs): They combine advantages of different materials (e.g., PEO+LLZO, PVDF+LATP) to enhance mechanical strength and ionic conductivity [44, 45]. Despite significant progress, the widespread application of solid‐state batteries faces challenges, including sluggish ion transport kinetics at solid‐solid interfaces, insufficient chemical/electrochemical compatibility, and constraints related to material cost and scalable manufacturing [46, 47, 48, 49].

Among various SSE materials, Metal‐Organic Frameworks (MOFs)—porous crystalline hybrid materials constructed from metal nodes and organic ligands—have recently emerged as promising candidates for energy storage and conversion [50, 51, 52, 53, 54]. Their advantages for SSEs include: (1) Tunable Pore Size and Chemistry: Precise selection of metal nodes and organic ligands allows for tailored pore size (0.3–3 nm) and surface functional groups, creating directional channels for rapid Li^+^/Na^+^ transport; (2) High Porosity and Active Sites: High specific surface area and open pore structures can effectively adsorb electrolytes or dissociate lithium salts, forming continuous ion conduction networks; (3) Chemical and Mechanical Stability: Certain MOFs exhibit excellent stability across wide voltage ranges and high temperatures, suitable for high‐voltage cathodes and lithium‐metal anodes. Their flexible frameworks can accommodate volume changes during cycling, inhibiting crack formation at interfaces [55]. Recent research on MOF‐based SSEs has focused on: (1) Intrinsic MOF Electrolytes: Achieving room‐temperature ionic conductivity >10^−4^ S cm^−1^ was realized by encapsulating lithium salts (e.g., Lithium bis (tri‐uoromethanesulphonyl) imide (LiTFSI)) within MOF pores [56, 57]; (2) MOF Composite Electrolytes: Combining MOFs with polymers (e.g., PEO, PVDF) or inorganic electrolytes (e.g., LLZO) to enhance Li^+^ transference number (>0.6) and suppress dendrite growth via a domain‐limiting effect [58, 59, 60]; and (3) Interfacial Functionalization: Modifying MOF surfaces with flexible polymers to reduce interfacial impedance and enhance cycling stability. Nevertheless, practical application of MOF‐based SSEs faces hurdles: (1) ionic conductivity of most MOFs remains lower than sulfide electrolytes (10^−2^ S cm^−1^); (2) hygroscopicity may lead to structural degradation, necessitating humidity‐tolerant systems; and (3) high grain boundary resistance and scalable production costs require optimization.

Conventional 3D MOF‐based electrolytes face intrinsic limitations, including tortuous ion conduction paths, poor interfacial contact due to rigid solid‐solid contact, and insufficient mechanical properties, often leading to brittleness [61, 62, 63]. First, the intricate and often disordered pore networks within 3D MOFs can lead to highly convoluted ion conduction paths. Lithium ions are forced to navigate these complex channels, frequently encountering spatial hindrance and strong interactions with pore walls. This not only increases the energy barrier for ion migration but also significantly reduces ionic conductivity, as demonstrated in 3D MOFs with complex topologies [64, 65, 66]. Second, the rigid nature of most 3D MOFs results in poor solid‐solid contact with electrodes. This incompatibility leads to gaps and non‐uniform interfaces, which in turn cause high interfacial resistance and inefficient charge transfer, ultimately degrading the battery's cycling performance and rate capability [67, 68]. Third, the intrinsic brittleness of many crystalline 3D MOFs compromises their mechanical integrity. They are susceptible to fracture or deformation during cell assembly and operation, posing a threat to the long‐term stability and safety of the battery [69, 70].

**FIGURE 1:**
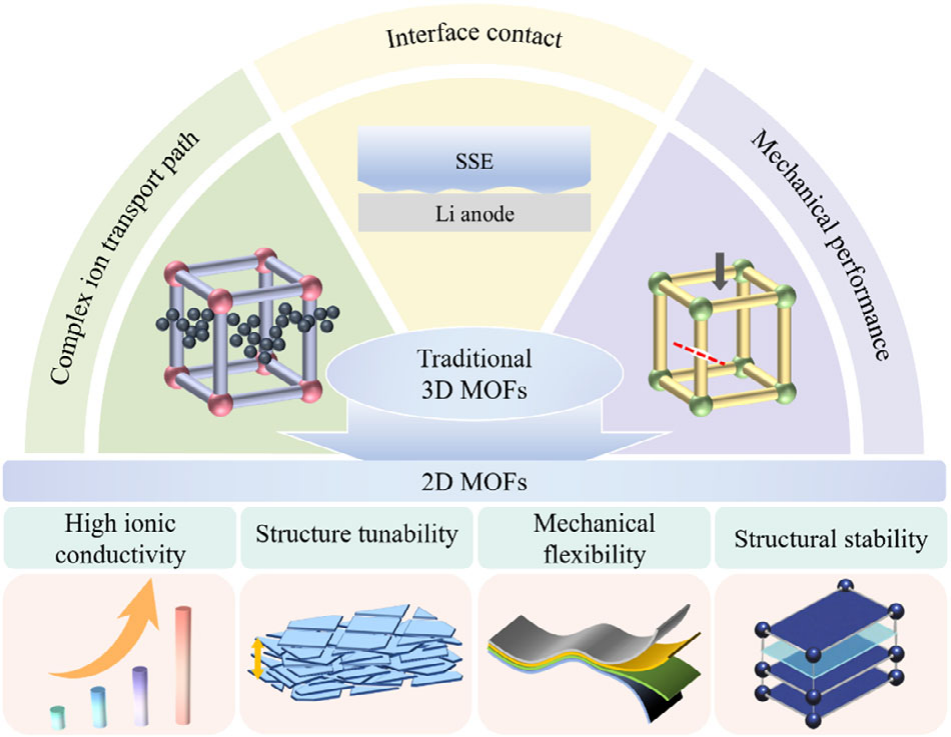
Comparison of 3D MOFs with 2D MOFs.

In contrast, 2D MOFs, an emerging branch of the MOF family, present unique advantages due to their ultrathin layered structure, which provides short, planar ion transport channels and abundant exposed active sites [71, 72, 73, 74, 75]. The strong interaction between unsaturated metal sites on the surface and polymer electrolytes can significantly enhance interfacial stability and ion transport efficiency. This work comprehensively reviews recent advances in 2D MOF‐based SSEs. We begin by elucidating the intrinsic advantages of 2D MOFs for electrolyte applications, followed by a discussion of their synthetic methodologies, structural characteristics, and composite strategies. Subsequent sections examine ion transport mechanisms and performance optimization, highlighting representative material systems and applications. Finally, we address current challenges and outline future research directions for this promising class of materials. Figure 1.

## 2 Advantages of 2D MOFs

2D MOFs offer distinct solutions to the limitations of conventional 3D MOFs, leveraging their intrinsic structural characteristics to provide outstanding advantages for solid‐state electrolytes. As evident from Table 1, a performance comparison between 2D MOFs and 3D MOFs can be observed.

**TABLE 1:** Performance comparison of composite solid‐state electrolytes based on 2D and 3D MOFs.

Filler type	Filler material	Matrix material	Ionic conductivity @RT (mS cm^−1^)	$t_{Li^{+}}$	Current density, (mA cm^−2^) cycle life (h)	Cathodes	Capacity after cycling (mA h g^−1^)	Refs.
2D MOFs	Cu‐BTC	PEO	0.046	0.58	0.1, 1300 at 60°C	LiFePO_4_	162 (500th cycle at 0.5C)	[81]
	Co‐BDC	PVDF	0.626	0.70	0.1, 750	LiFePO_4_	140 (650th cycle at 0.5C)	[77]
	Cu‐HHTC	PVDF‐HFP	1.59	0.81	0.1, 3200	LiFePO_4_	115.4 (1000th cycle at 1C)	[99]
	Cu (BDC‐2OH)	—	0.776	0.81	0.2, 1000 at 0°C	LiFePO_4_	171.2 (300th cycle at 0.5 C)	[84]
3D MOFs	ZIF‐67	PEO‐SN	0.117	0.40	0.1, 550 at 30°C	LiFePO_4_	∼65 (300th cycle at 1 C)	[100]
	ZIF‐8	PVDF	0.408	0.64	0.1, 1500	LiFePO_4_	146.6 (500th cycle at 0.5 C)	[101]
	MIL‐125‐Li	PVDF	0.39	0.54	0.1, 700 at 28°C	LiFePO_4_	130 (500th cycle at 0.5 C)	[69]
	UIO‐66	—	0.32	0.33	0.2, 100 at 60°C	LiFePO_4_	112 (380th cycle at 1 C)	[56]

### 2.1 High Ionic Conductivity

2D MOFs can achieve high ionic conductivity, offering a distinct advantage over their 3D counterparts. In 3D MOFs, Li^+^ ions must navigate long, tortuous pathways through a continuous pore network, resulting in high migration resistance. Furthermore, their relatively narrow pores are susceptible to blockage by polymer chains or lithium salts, further impeding ion transport. In contrast, 2D MOFs provide short, planar transport pathways either across the nanosheet surfaces or through the interlayer galleries, enabling rapid Li^+^ migration. The extensive interfaces formed between the large lateral surfaces of 2D MOFs and polymer matrices can also serve as efficient highways for Li^+^ conduction. Collectively, these attributes contribute to significantly higher ionic conductivity when 2D MOFs are incorporated as fillers in solid‐state electrolytes.

Exemplifying this principle, a series of 2D anionic frameworks named Ti‐DMTHA‐M have been developed based on a titanium catecholate system. These materials are constructed from Ti(C_2_O_2_)_3_ metal centers and anthracene‐based ligands, specifically 9,10‐dihydro‐2,3,6,7‐tetrahydroxyanthracene (DMTHA), forming a net‐like structure with hcb topology. The cations residing within the pores of Ti‐DMTHA‐M play a critical role in solid‐state ion conduction, and the ionic conductivity of this series demonstrates a progressive, tunable increase [76]. These 2D MOFs not only defy the conventional perception of inefficient interlayer transport but also establish continuous ion‐conducting channels, achieving conductivity that markedly surpasses that of traditional 3D MOFs.

### 2.2 Structure Tunability

#### 2.2.1 Shortened Ion Transport Paths

The ultrathin nature of 2D MOFs inherently shortens ion diffusion distances. For example, 2D cobalt‐based MOF nanosheets (CMS) can achieve an average thickness of just 0.3 nm [77]. Morphological engineering, such as the development of petal‐like Ni‐MOF nanosheets, substantially increases the accessible surface area, creating dense networks for accelerated interfacial ion transfer [78]. Introducing the 4‐n‐butylbenzoic acid (NBA) modifier, the flower‐like 2D MOFs material CuBDC‐10 with thin nanosheet stacking can be prepared, and its 2D lamellar structure accelerates the hopping transport of Li^+^ along the 1D channels, which significantly shortens the Li^+^ transport path [79].

#### 2.2.2 In‐Plane Ordered Pores

Many 2D MOFs feature periodically arranged intra‐layer pores (e.g., hexagonal honeycomb or square pores) that provide well‐defined, fast transport channels for ions like Li^+^ and Na ^+^ [80]. For example, Cu‐BTC, with its hexagonal lattice structure, allows Li^+^ to migrate rapidly along the centers of benzene rings, which form the apexes of the honeycomb pore channels [81]. The 1D ordered pores within 2D Cu(BDC) facets create a direct, fast Li^+^ transport path [82].

#### 2.2.3 Abundant Surface Functional Groups

Functional groups on 2D MOFs can promote ion dissociation and transport. The electron‐donating group ‐NH_2_ can restrict anion movement, thereby enhancing cation transport [83]. The hydroxyl groups (‐OH) on the framework surface of MOF‐2OH@DMF(Li) can form hydrogen bonds with the C═O groups of the N, N‐Dimethylformamide (DMF) molecules. Under the combined effect of nanoconfinement, this interaction induces the rearrangement of DMF molecules along the internal channels of the MOF, resulting in the formation of a long‐range ordered, continuous, and stable solvent layer. This well‐ordered DMF solvent layer can serve as “highways” for Li^+^ transport, effectively reducing the Li^+^ migration energy barrier. Concurrently, hydrogen bonding weakens the binding affinity between the C═O groups of DMF and the Li^+^ ions, thereby facilitating Li^+^ desolvation and promoting its rapid migration [84].

#### 2.2.4 Exposed Active Sites

Compared to 3D MOFs, 2D MOF nanosheets expose a greater number of metal centers and functional groups on their surfaces and pore walls, thereby enhancing interactions with the external environment. Furthermore, their unsaturated coordination bonds, ultrathin structure, and high porosity facilitate faster mass transport. These combined properties are crucial for applications in solid‐state electrolytes. Notably, thinner 2D MOF nanosheets possess a higher surface‐atom ratio, leading to an increased density of exposed active sites [85].

### 2.3 Superior Mechanical Flexibility

The ultrathin layered structure of 2D MOFs imparts excellent mechanical flexibility, a stark contrast to the often rigid and brittle nature of 3D MOFs. This flexibility enables 2D MOF‐based solid electrolytes to form intimate, conformal contact with electrode surfaces [86, 87, 88]. For instance, a 2D porphyrin MOF assembled from zinc nodes and flexible linkers exhibit good folding capability and mechanical flexibility [89]. This property ensures stable performance under bending and stretching in flexible devices [90], effectively improving interfacial contact and reducing interfacial resistance—a key bottleneck for traditional solid‐state electrolytes.

### 2.4 Enhanced Structural Stability

Many 2D MOFs—particularly those featuring strong coordination bonds such as metal‐sulfur and metal‐nitrogen bonds, along with extended π‐π stacking interactions—exhibit superior mechanical and chemical stability compared to conventional MOFs and other related materials [91, 92, 93]. The structural stability of 2D MOFs is closely associated with the interactions between functional groups and metal centers. Functional groups such as ―NH_2_, ―SH, and ―SeH can further enhance thermal and chemical stability by forming stronger chemical bonds [94]. In addition, certain bidentate ligands can serve as pillars that connect chiral corrugated layers, reinforcing coordination with metal centers. This configuration helps resist temperature variations and mechanical stress, thereby preserving structural integrity and contributing to excellent thermal and mechanical stability [95]. These design features enable 2D MOFs to demonstrate superior stability compared to conventional 3D MOFs under specific conditions, including neutral or weakly acidic media, low‐to‐medium voltage windows, appropriate temperature ranges (−20°C–100°C), and non‐aggressive redox environments.

It is worth emphasizing that the structural stability and mechanical flexibility of 2D MOFs are not mutually exclusive; rather, they are designed to achieve a “rigid‐flexible hybrid” property. Specifically, their excellent structural stability endows 2D MOFs with superior chemical and thermal stability, enabling them to resist structural collapse during electrochemical cycling or in high‐temperature environments. Meanwhile, their 2D layered structure confers good mechanical flexibility to the material without compromising the rigidity of the interlayer framework. This “rigid‐flexible hybrid” property plays a crucial role in suppressing lithium dendrites. On one hand, interlayer flexibility allows 2D MOF‐based electrolytes to tightly conform to the rough surface of lithium metal anodes, inhibiting dendrite nucleation. On the other hand, the in‐layer rigid framework acts as a physical barrier, preventing lithium dendrites from penetrating the electrolyte [96, 97]. Furthermore, this dual‐rigidity‐flexibility property enables the electrolyte to dynamically accommodate the volumetric changes of lithium metal during charge‐discharge cycles, maintaining the integrity of ion transport pathways. This prevents interfacial cracking and subsequent dendrite growth along crack pathways [98]. This “rigid‐flexible hybrid” mechanism bridges the performance gap between the poor interfacial contact of traditional rigid inorganic electrolytes and the insufficient mechanical strength of flexible polymer electrolytes, offering a unique solution for the stable operation of lithium metal solid‐state batteries.

## 3 Structural Characterization and Preparation of 2D MOFs

### 3.1 Structural Characterization

2D MOFs are layered porous materials formed by the coordination of metal ions/clusters with organic ligands, creating highly ordered 2D networks. Their key structural features include:

#### 3.1.1 Atomic‐Level Thickness and Layered Structure

Single or Few‐Layer Stacking: 2D MOFs typically comprise single or few‐layer lamellae, characterized by strong in‐plane coordination bonds and weak interlayer interactions—such as van der Waals forces, π‐π stacking, or hydrogen bonding. These weak interlayer forces not only afford structural flexibility but also facilitate ion intercalation. For instance, Ni_3_HIB_2_ features interlayer channels that enable rapid Li^+^ diffusion, supporting high‐capacity energy storage [102]. The porous nature of 2D MOFs further makes them promising candidates as solid‐state electrolytes, allowing efficient lithium‐ion transport between layers.

Within 2D planes, a large π‐conjugated system enhances structural stability. Planar conjugated ligands—such as benzene, triphenylene, and phthalocyanines—form extended π‐conjugated networks through metal‐ligand coordination. This conjugation strengthens in‐plane bonding and improves structural integrity. Moreover, symmetric ligands often generate hexagonal or honeycomb frameworks, which are further stabilized by π‐conjugation, contributing to the overall robustness of the 2D structure [103].

Lateral Extensibility: The in‐plane structure of 2D MOFs can be extended to a micrometer scale, enabling the fabrication of large‐area thin films or freestanding lamellae. Using advanced interfacial synthesis strategies—such as coordination polymerization and condensation—defect‐free, highly crystalline, and large‐area 2D MOF films have been successfully prepared. These can be exfoliated into free‐standing nanosheets with ultrahigh aspect ratios up to 2000:1 and thicknesses as low as ∼1.7 nm. Notably, the pore structures in such nanosheets are clearly resolvable via high‐resolution transmission electron microscopy (HRTEM) at near‐atomic resolution. These results confirm that highly ductile and ultrathin 2D MOF layers can be reproducibly fabricated through tailored synthesis routes [104].

#### 3.1.2 Highly Ordered Pore Structure

Metal nodes and organic ligands form periodically arranged intralayer pores (e.g., hexagonal/honeycomb, square/rhombic). Pore size and shape can be precisely tuned by varying ligand length or metal cluster size, with tunable layer spacing (0.3–2 nm) accommodating different ion sizes. For example, Cu‐OHDDQP assembles into a novel quasi‐honeycomb lattice topology through the distinct coordination geometry between nonplanar D2‐symmetric conjugated ligands (OHTPPQ) and Cu (II) nodes [105], the intralayer connectivity and interlayer stacking pattern of mos‐MOF is hexagonal pores [106], and CuBDC‐NO_2_ nanosheets in AB_0.17_ stacking pattern are rhombic pores [107]. Layer spacing can also be modulated by intercalating atoms/solvents or attaching alkyl chains of varying lengths to ligands [108]. These pores and Layer spacing are regularly arranged to form a highly ordered structure, facilitating lithium ion transport.

#### 3.1.3 Surface Functionalization Diversity

Physicochemical properties can be tailored via: (1) Ligand Pre‐functionalization: Modifying organic ligands before synthesis to introduce target functional groups (e.g., pyridine, thiophene) [109, 110]. (2) Post‐Synthetic Modification (PSM): Utilizing active sites on MOF ligands (unsaturated metal sites, active functional groups) for subsequent reactions (e.g., introducing hydroxyl groups via click chemistry) [111]. (3) Metal Node Modification: Grafting other metal ions or organic molecules onto metal nodes (e.g., synthesizing bimetallic 2D MOFs like CoFe‐BDC) [83].

#### 3.1.4 Defects and Edge Effects

Defects and edge sites in 2D MOFs play a crucial role in governing their physicochemical properties and functional behavior when applied as solid‐state electrolytes. Rather than being merely structural imperfections, defects—including vacancies, dopants, and lattice distortions—can introduce new active sites that enhance ion transport and interfacial properties. Meanwhile, the edges of exfoliated 2D MOF nanosheets often expose unsaturated metal centers and coordinatively unsaturated ligands, which serve as highly reactive sites. These structural features can significantly modulate the overall electrochemical behavior of solid‐state electrolytes.

On the one hand, certain defect types—such as metal node vacancies or ligand deficiencies—can create additional pathways for ion migration, thereby increasing ionic conductivity. The presence of defects may also facilitate stronger interfacial adhesion with electrodes, improving mechanical integrity and reducing interfacial resistance. On the other hand, the exposed metal sites at nanosheet edges often function as active centers for Lewis acid‐base interactions. These interactions can assist in regulating ion flux, promoting uniform lithium or sodium deposition, and suppressing dendrite formation. Moreover, edge‐terminated functional groups (e.g., ―OH, ―COOH, or ―NH_2_) can participate in hydrogen bonding or electrostatic interactions with polymer matrices or inorganic solid electrolytes, further enhancing the structural stability and cyclability of the composite electrolyte.

Importantly, the combination of defect‐induced active sites and edge‐mediated reactivity provides a synergistic mechanism for tailoring ion transport and interfacial properties in all‐solid‐state batteries. For instance, defect‐rich and edge‐exposed 2D MOFs have shown improved wettability and compatibility with lithium metal anodes, contributing to more stable solid electrolyte interphases (SEI). However, excessive defects or uncontrolled edge reactivity may also introduce electronic conduction or cause undesirable side reactions. Therefore, precise control over defect type, density, and edge chemistry through synthetic or post‐synthetic strategies is essential to harness these structural features for high‐performance, durable solid‐state electrolytes.

Future research should focus on correlating specific defect types and edge configurations with ion transport mechanisms and interfacial behavior, using advanced characterization techniques such as scanning transmission electron microscopy, synchrotron X‐ray absorption spectroscopy, and solid‐state nuclear magnetic resonance (SSNMR). Such fundamental understanding will pave the way for defect and edge engineering of 2D MOFs as next‐generation solid electrolyte materials.

### 3.2 Preparation Techniques

The choice of synthesis method is crucial for achieving precise structural control in 2D MOFs. Common techniques include:

#### 3.2.1 Solvothermal/Hydrothermal Synthesis

**FIGURE 2:**
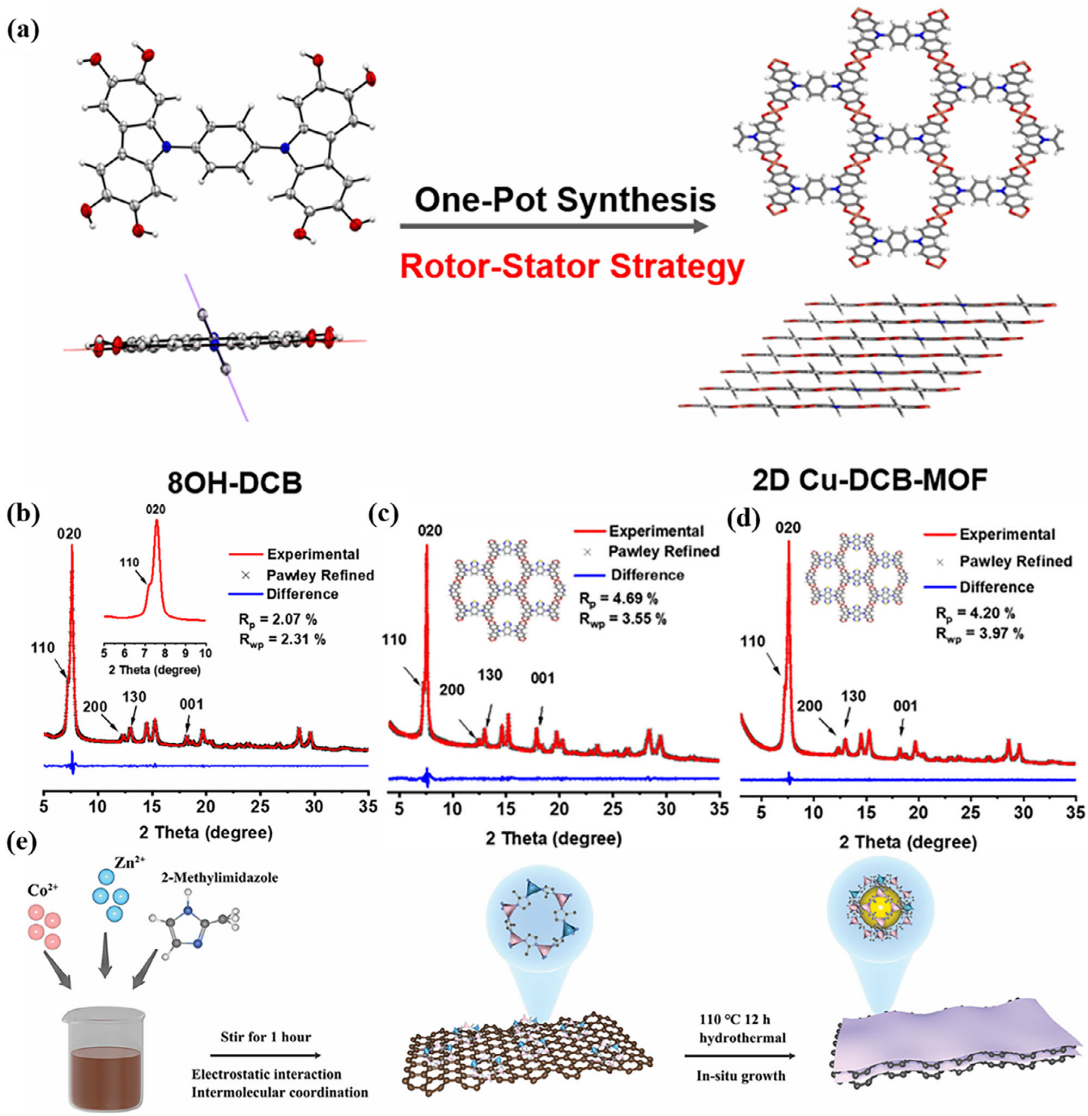
a) Synthesis of Cu‐DCB‐MOF from the 8OH‐DCB ligand. Experimental (red, Cu Kα1), simulated PXRD patterns (black dotted line) of b) Cu‐DCB‐MOF, c) Cu‐DCBT‐MOF and d) Cu‐DCBBT‐MOF and their difference plot (blue). a‐d) Reproduced with permission [112]. Copyright 2024, American Chemical Society. e) Preparation of Co_x_Zn_1−x_‐MOF/rGO by hydrothermal synthesis. Reproduced with permission [114]. Copyright 2025, Wiley‐VCH.

Solvothermal/hydrothermal synthesis facilitates the self‐assembly of metal ions and organic ligands into layered structures within a sealed reactor under elevated temperature and pressure. In this process, metal salts and ligands are dissolved in organic solvents (e.g., DMF, ethanol), where the solvent acts not only as a reaction medium but also as a critical regulator of solubility, coordination environment, and crystal growth kinetics. This regulation promotes the oriented linkage of metal ions and ligands, leading to the in‐plane extension of a 2D network. The resulting layers are stabilized by interlayer van der Waals forces, π‐π stacking, and other weak interactions. For instance, 2D MOFs with copper centers have been synthesized via solvothermal reaction using nonplanar biligands such as 8OH‐DCB, 8OH‐DCBT, 8OH‐DCBBT, and 8OH‐TPB (Figure 2a–d) [112, 113]. In another example, a coordination reaction between 2‐methylimidazole and Co^2+^ and Zn^2+^ on a graphene oxide (GO) surface under hydrothermal conditions induced the in situ growth of an ultrathin 2D MOF structure on GO (Figure 2e) [114].

This method is renowned for its mild conditions and controllable crystal growth. Key parameters—including solvent selection, reaction temperature, and ligand structure—enable precise tuning of the interlayer spacing, pore architecture, and crystallinity of the resulting 2D MOFs. As one of the most prevalent synthesis strategies (Table 2), it provides a fundamental and versatile platform for designing functionalized 2D MOF materials.

**TABLE 2:** Solvothermal Synthesis of 2D MOF Materials.

2D MOF	Solvent	Temperature (°C)	Time (h)	Thickness (nm)	Refs.
NUS‐8(Zr/ Hf)	H_2_O	120	20	10–20	[115]
Ni‐Fe‐MOF NSs	DMAC/ H_2_O	150	—	1.67–2.58	[116]
Ni‐M‐MOF NSs (M = Co, Mn, Zn, Cd)	DMAC/ H_2_O	150	—	—	[116]
MF‐ZrBTB	DMF/HCOOH	130	0.53	∼3	[117]
ST‐ZrBTB	DMF/HCOOH	130	96	∼45	[117]
CuCo‐BDC	H_2_O	25	24	—	[118]
Co MOF‐Py_3_	DMF/ Py	120	48	∼2.8	[119]

#### 3.2.2 Mechanical/Chemical Exfoliation

Chemical exfoliation primarily utilizes intercalation reagents, including surfactants or organic solvents, which preferentially insert between the MOF layers. This insertion chemically weakens interlayer cohesion or increases the interlayer spacing, facilitating separation. For instance, ultrathin Cu‐TCPP nanosheets can be prepared with the surfactant aid of polyvinylpyrrolidone (PVP) [120]. In another approach, 4,4’‐dipyridyl disulfide (DPDS) acts as an intercalating agent, where its pyridine nitrogen atoms coordinate with metal centers and pry the layers apart, resulting in new ultrathin 2D MOF crystals [121].

**FIGURE 3:**
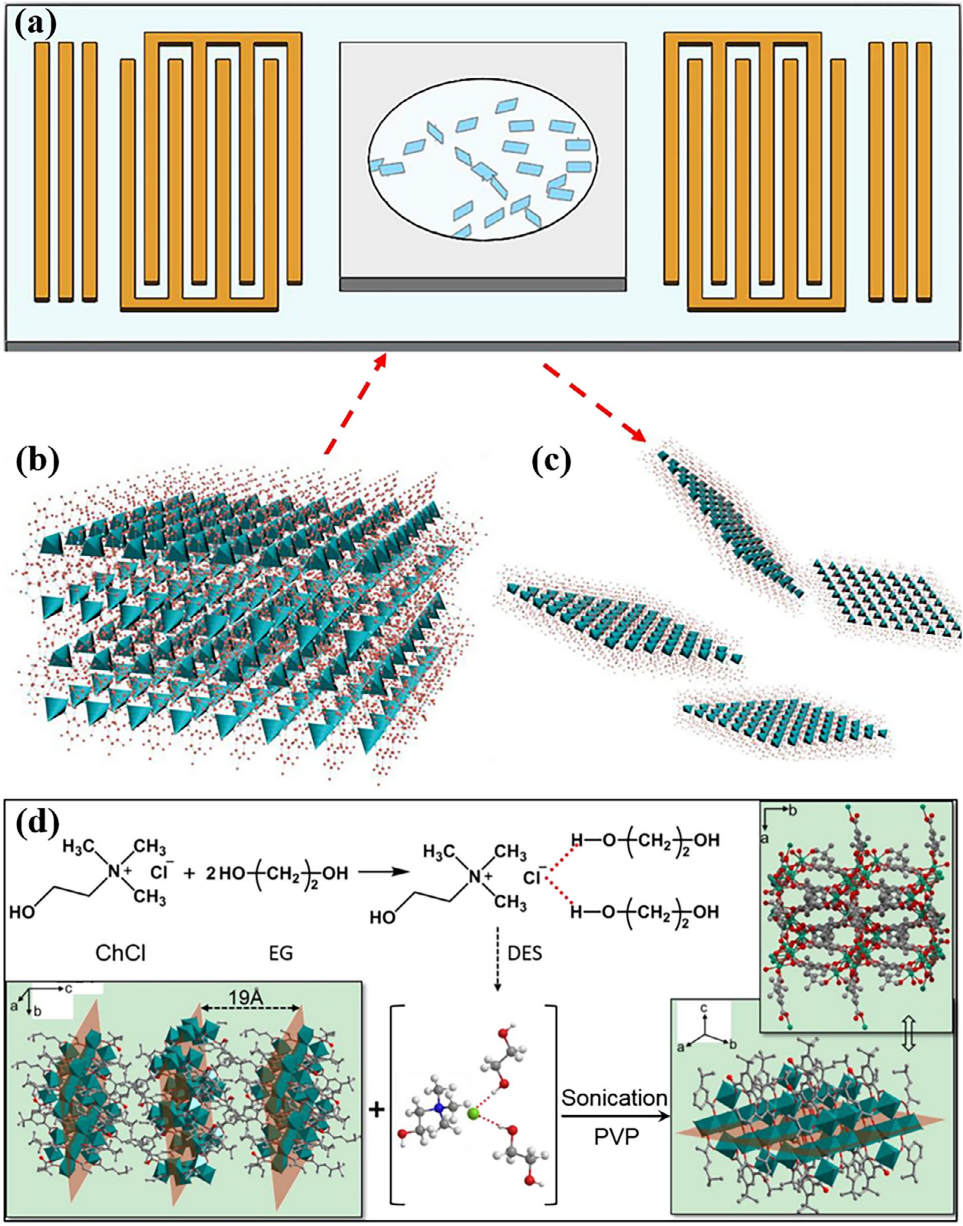
a) Illustration of the SAW exfoliation device. b) Illustration of the native Zn_2_(bim)_4_ structure. c) Illustration of a single‐layer Zn_2_(bim)_4_ nanosheet structure. a‐c) Reproduced under the terms of the CC‐BY‐NC‐ND license [124]. Copyright 2022, Elsevier. d) MAMS‐1 exfoliated in DES into fewer layers of MAMS‐1‐NS. Reproduced under the terms of the CC‐BY‐NC‐ND license [126] Copyright 2021, Elsevier.

In contrast, mechanical exfoliation employs external physical forces—such as grinding, ball milling, or ultrasonication—to directly overcome interlayer interactions [122]. A representative example is the production of large‐area Zn_2_(bim)_4_ nanosheets via direct sonication of its bulk counterpart [123]. Beyond conventional methods, innovative techniques have been explored. Surface acoustic wave (SAW) technology, for example, utilizes an electric field to achieve rapid, bulk exfoliation, although it typically yields very small nanosheets with a low monolayer ratio (Figure 3a–c) [124]. Adhesive tape‐based methods can transfer layers to substrates like silicon wafers, but the weak coordination in some MOFs makes them prone to damage during this process, compromising product integrity [125]. Solvent‐assisted liquid‐phase exfoliation is another effective variant. The deep eutectic solvent (DES), for instance, has been used to exfoliate the bulk material MAMS‐1 into structurally intact nanosheets (MAMS‐1‐NS), with the principle illustrated in Figure 3d [126]. Similarly, ultrasonic treatment of the precursor Co(CNS)_2_(pyz)_2_ dispersed in ethanol yielded powdered monolayer nanosheets without disrupting the intralayer crystal structure [86]. A significant challenge for mechanical exfoliation is the requirement for precise control and extensive optimization of parameters. Without this, the process often suffers from very low yields. Furthermore, achieving uniform layer separation is difficult; it is frequently accompanied by structural damage or nanosheet re‐aggregation, which further diminishes the effective yield.

In summary, both mechanical and chemical exfoliation methods work by strategically targeting the weak forces between 2D MOF layers. The resulting exfoliated nanosheets largely retain the original MOF's intralayer coordination structure and functional active sites, providing a crucial material foundation for constructing advanced nanocomposites and flexible devices.

#### 3.2.3 Interfacial Synthesis

Interfacial synthesis enables the direct assembly of 2D MOF films through a controlled reaction between metal ions and organic ligands at gas/liquid or liquid/liquid interfaces.

**FIGURE 4:**
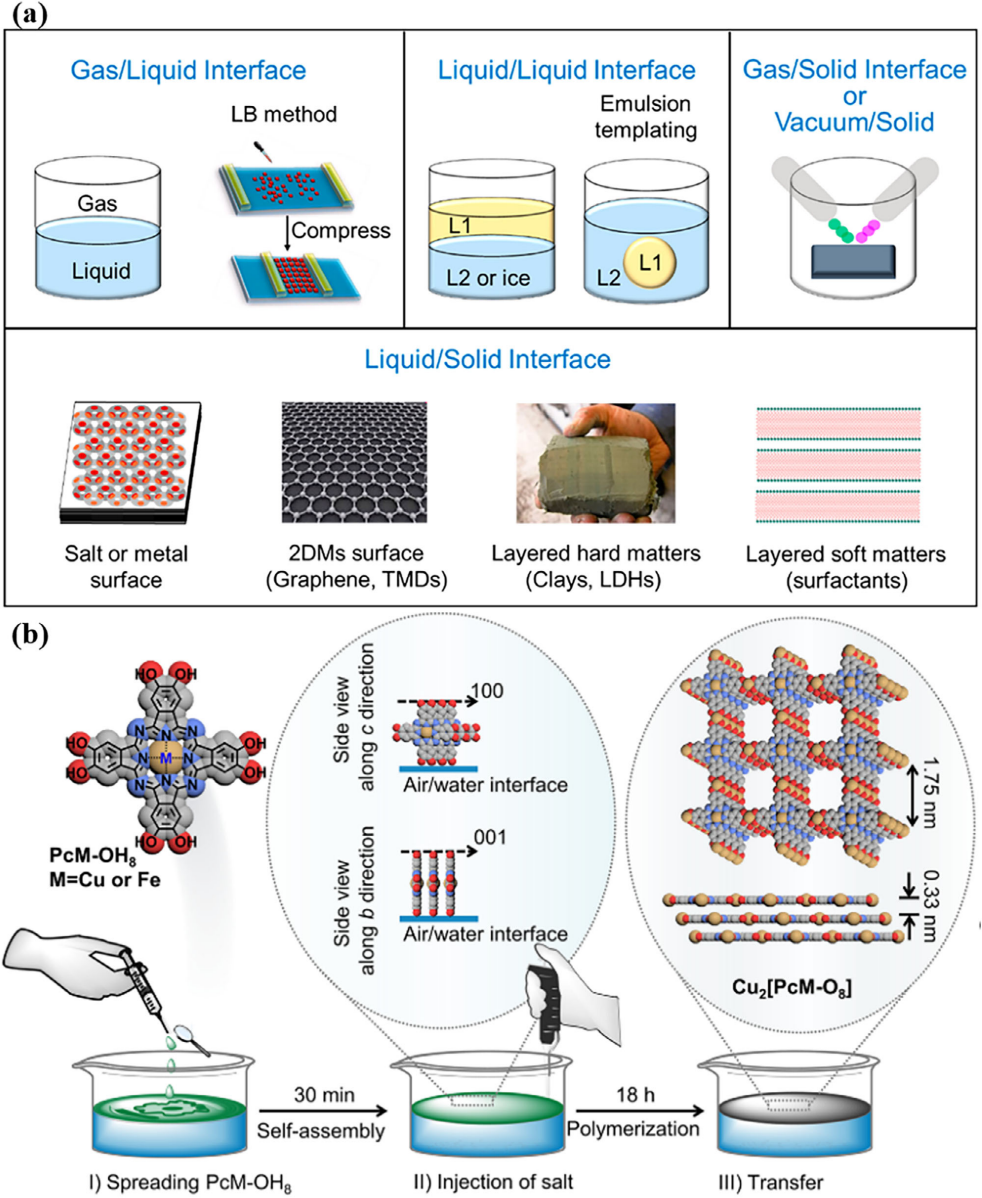
a) Schematic illustration of various interfaces for chemical synthesis, including gas/liquid, liquid/liquid. Reproduced with permission [129]. Copyright 2018, American Chemical Society. b) Preparation of MOF Cu_2_(PcM‐O_8_) and atom modeling by interfacial synthesis. Gray, red, blue and white spheres represent Cu, O, N, and C atoms, respectively. Reproduced with permission [127]. Copyright 2021, American Chemical Society.

This strategy facilitates the in‐situ formation of 2D MOF films by leveraging directional diffusion and confined coordination reactions at the interface (Figure 4a). The 2D constrained environment inherently restricts crystal growth to the interfacial plane, effectively suppressing the formation of 3D bulk phases. A representative example is the interlayer‐directed growth of the 2D MOF Cu_2_(PcM‐O_8_) at an air‐water interface. As shown in Figure 4b, this process is driven by the π‐π interactions of phthalocyanine molecules and modulated by hydrophobic forces, resulting in a perpendicular alignment of the metal‐phthalocyanine ligands at the water surface [127]. Beyond conventional gas‐liquid and liquid‐liquid systems, surfactant bilayer templating has emerged as a powerful extension of this concept, enabling the synthesis of sub‐10‐nm 2D MOF nanosheets [128]. This approach utilizes surfactant assemblies to physically confine vertical MOF growth while simultaneously providing colloidal stabilization for the resulting nanosheets [129].

Benefiting from the spatial confinement and modulated diffusion kinetics at the interface, this method allows for the direct preparation of large‐area, highly oriented, and ultrathin MOF films with precise control over thickness and structural integrity. The resulting films successfully retain the intrinsic intralayer coordination networks and accessible active sites characteristic of 2D MOFs.

#### 3.2.4 Vapor‐Phase Deposition

**FIGURE 5:**
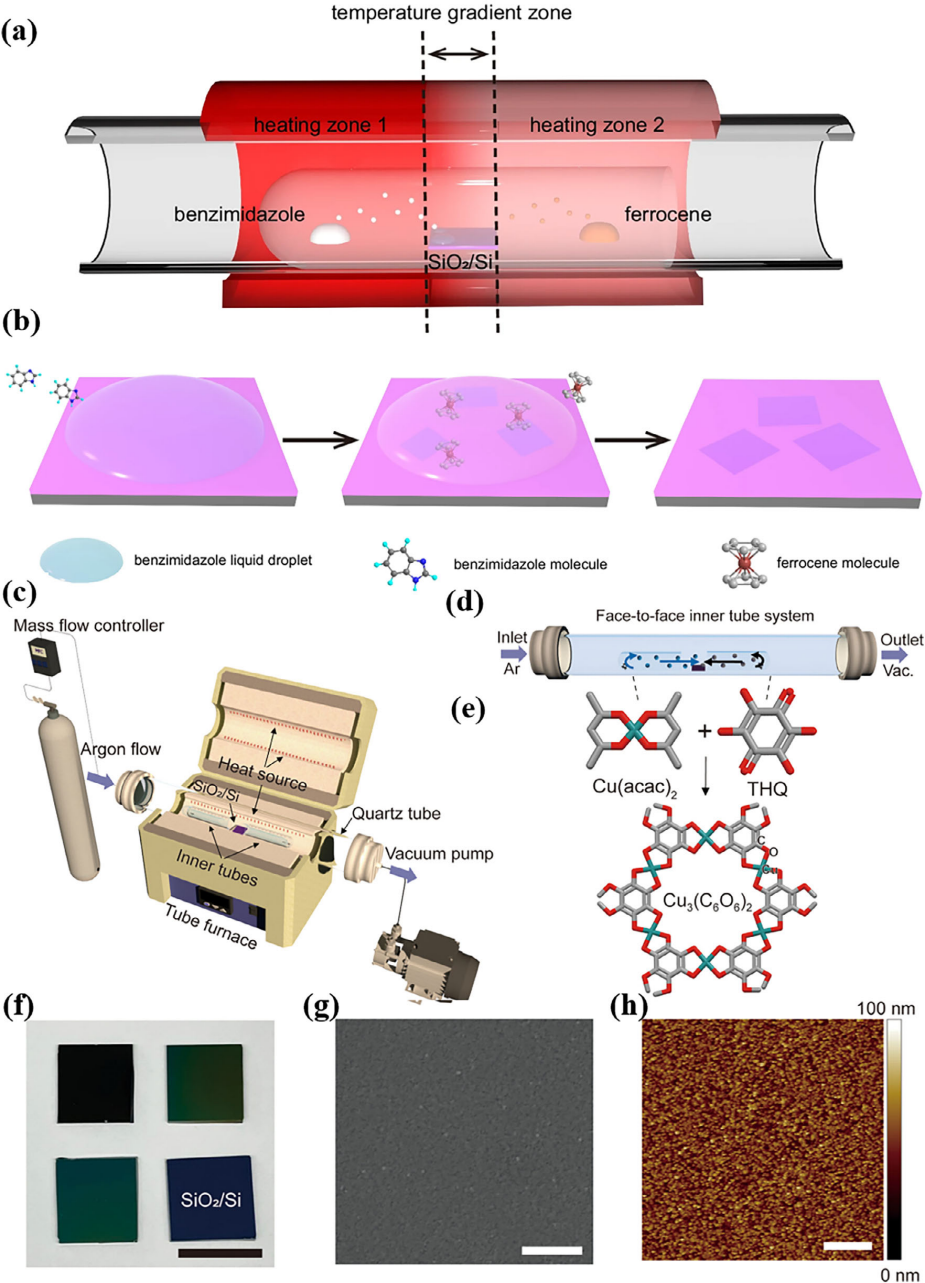
a) Schematic diagram of the Self‐condensation‐assisted CVD SCA‐CVD growth apparatus. b) The proposed SCA‐CVD growth method includes the formation of droplets due to the self‐condensation of benzimidazole precursor vapors, the dissolution and diffusion of evaporated ferrocene molecules in the droplets, and the formation of atomically thin single crystals of Fe_n_(bim)_2n_. a,b) Reproduced under the terms of the CC‐BY license [130] Copyright 2024, Springer Nature. Illustration of the c) overall and d) detailed face‐to‐face inner tube system setup for Cu_3_(C_6_O_6_)_2_ synthesis. e) Reaction scheme for the vapor‐phase reaction. f) Optical image (scale bar: 1 cm), g) SEM image (scale bar: 2 µm), and h) AFM image (scale bar: 2 µm) of the resulting Cu_3_(C_6_O_6_)_2_ thin films on a SiO_2_/Si substrate. c–h) Reproduced with permission [131]. Copyright 2022, American Chemical Society.

Vapor‐phase deposition (VPD) enables the in‐situ synthesis of highly crystalline, large‐area 2D MOF films via gas‐solid reactions between metal precursors and organic ligands on a heated substrate. In this process, volatile metal compounds and ligands are vaporized and transported by a carrier gas to a temperature‐controlled substrate. Upon adsorption, the gaseous molecules diffuse and undergo coordination reactions on the hot surface. By precisely regulating precursor vapor pressure, substrate temperature, and reaction time, the assembly of metal ions and ligands is directed along the substrate plane, resulting in a continuous 2D layered structure. For instance, a designed temperature gradient can be employed to sublimate the precursor, followed by condensation of the ligand into liquid droplets. Metal ions then dissolve and diffuse within these droplets, facilitating a coordination reaction that yields large‐area 2D MOF single crystals on the substrate (Figure 5a,b) [130]. In an alternative approach, highly crystalline 2D MOF films are directly synthesized at high temperatures through gas‐phase exchange reactions (Figure 5c–h) [131]. Another configuration involves placing the metal salt and organic ligand in separate heating zones. Under a nitrogen purge and low‐pressure environment, precursor evaporation is promoted, allowing metal and ligand vapors to migrate with the gas flow to the substrate surface. This triggers a coordination polymerization reaction that forces the ligands into a 2D order, enabling the controllable growth of ultra‐smooth 2D MOF films [132].

The elevated temperature environment serves multiple critical functions: it facilitates precursor decomposition and ligand activation, while thermodynamically optimizing crystal growth kinetics to suppress 3D agglomeration and promote planar epitaxial growth. As a solvent‐free technique, VPD allows for precise control over film thickness and crystallographic orientation, ultimately producing centimeter‐scale, uniform 2D MOF films with minimal defects.

#### 3.2.5 Electrochemical Preparation

Electrochemical synthesis enables the formation of 2D MOF films through the electrodeposition of metal salts and ligands onto an electrode surface, typically via constant potential or pulsed techniques. This method applies electrical signals to drive electrochemical reactions in solution, resulting in the in situ assembly of MOF films. The electrode functions dually as both the electron donor/acceptor and the growth substrate, facilitating the directional coordination of metal ions and ligands into planar, 2D layered structures under the applied potential.

**FIGURE 6:**
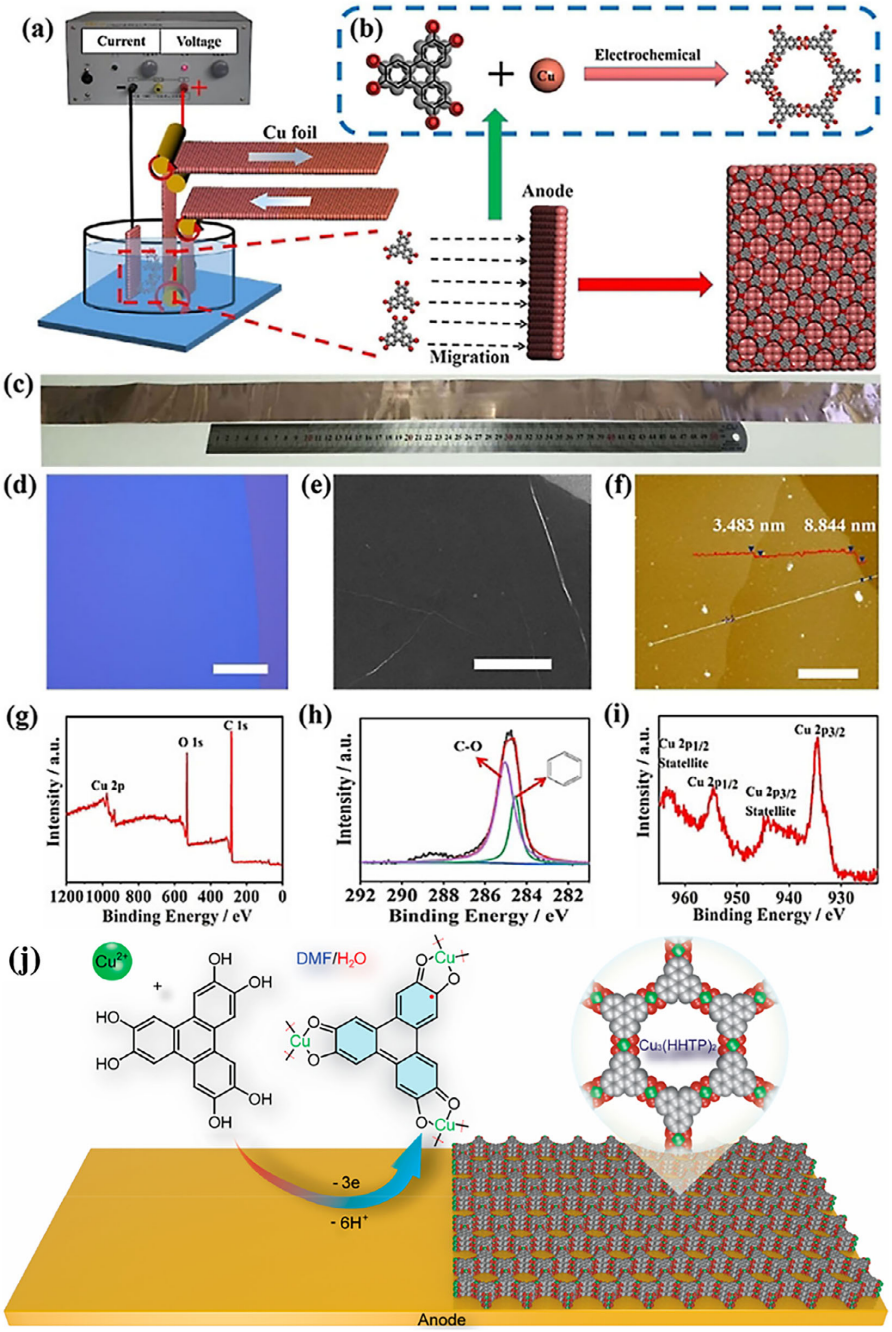
a) Electrochemical reaction cell for the preparation of a Cu_3_(HHTP)_2_ film on Cu foil. b) Schematic diagram of coordination reaction between Cu^2+^ and the HHTP ion. c) Optical image of the Cu foil with Cu_3_(HHTP)_2_ MOF film. d) Optical image of Cu_3_(HHTP)_2_ film. Scale bar 50 mm. e) SEM image of uniform Cu_3_(HHTP)_2_ film. Scale bar 10 mm. f) AFM image of Cu_3_(HHTP)_2_ film. Scale bar 5 mm. g) XPS spectra of Cu_3_(HHTP)_2_ film. h) High resolution C 1s and i) Cu 2p XPS spectra of Cu_3_(HHTP)_2_ film. a–i) Reproduced with permission [133]. Copyright 2020 Wiley‐VCH. j) Schematic diagram of potentiostatic electrochemical deposition for preparing Cu_3_(HHTP)_2_ thin films. Reproduced with permission [135]. Copyright 2023, American Chemical Society.

Metal ion sources for this process fall into two primary categories. The first involves the anodic oxidation of a metal electrode to generate ions in situ. For instance, using a copper foil as both anode and cathode in an ammonia solution allows ligands to deprotonate into anions. Under constant potential, the anode undergoes oxidation to release Cu^2+^ ions, which subsequently coordinate with the ligand anions to directly grow a 2D MOF film on the anode surface (Figure 6a–i) [133]. The second approach utilizes metal salts pre‐dissolved in the electrolyte, commonly within a standard three‐electrode system. By applying a constant potential for a controlled duration, a corresponding 2D MOF film is synthesized from the precursor solution via a static electrodeposition process (Figure 6j) [134, 135].

This electrochemical strategy offers precise control over critical parameters—including solvent composition, electrolyte type, and applied potential—which directly govern the crystallinity, interlayer spacing, and electrode adhesion of the resulting MOFs. Characterized by mild conditions, short reaction times, real‐time monitoring capability, and inherent scalability, anodic deposition in particular demonstrates significant promise for the large‐scale production of 2D MOF films [136].

### 3.3 Structural and Preparation of 2D MOF Complex Electrolytes

**FIGURE 7:**
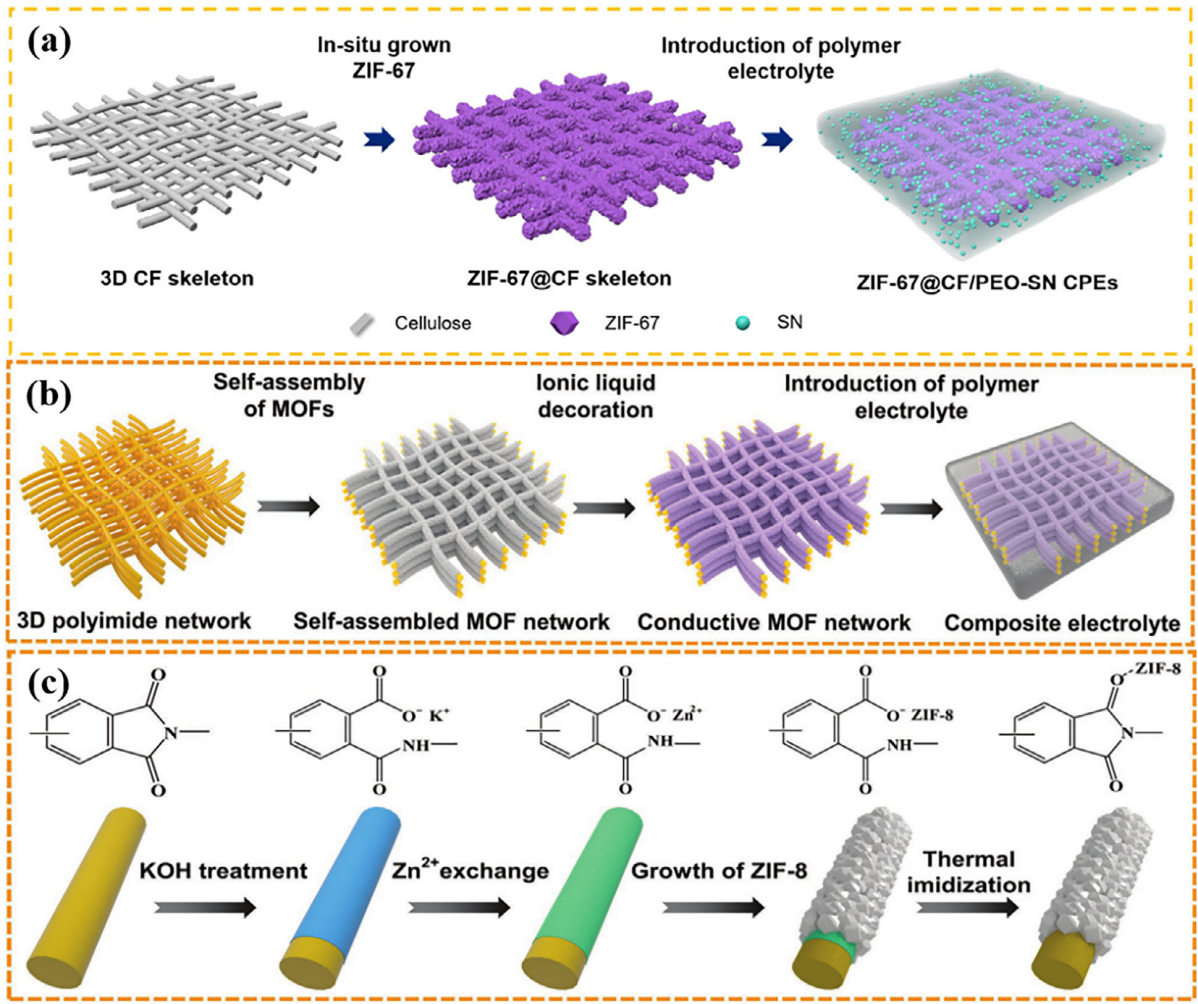
a) Schematic diagram of the preparation process of ZIF‐67@CF/PEO‐SN CPEs. Reproduced with permission [100]. Copyright 2024, American Chemical Society. b) Schematic diagram of preparation process of the composite solid electrolyte based on hierarchically self‐assembled MOF network. c) Schematic of the self‐assembly process. b,c) Reproduced with permission [101]. Copyright 2022, Wiley‐VCH.

**FIGURE 8:**
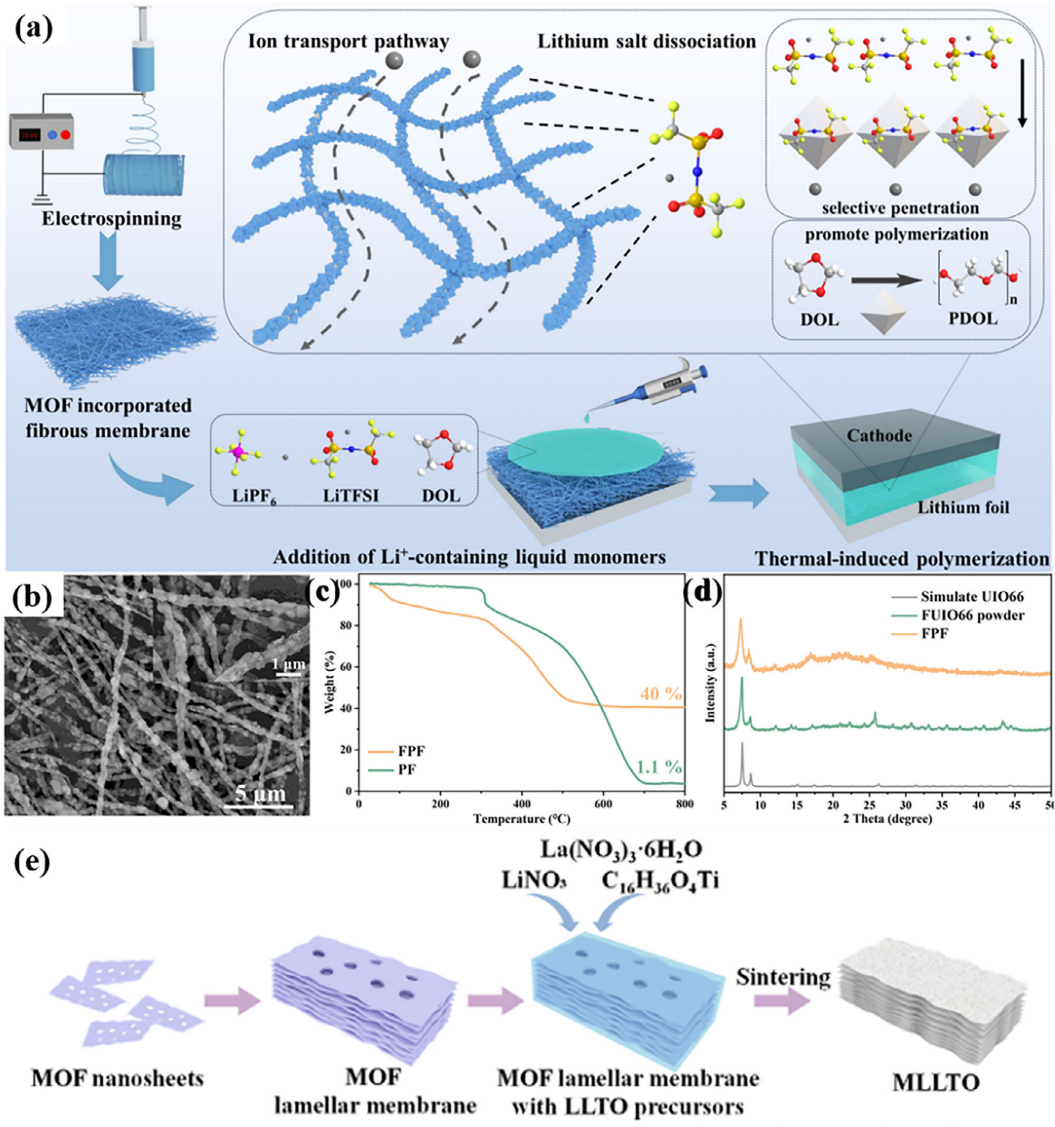
a) Design and fabrication principles of FPF@PDOL. b) SEM images of an interconnected 3D‐FUIO66/PAN nanofibers. c) TG curves of FPF and PF. d) XRD patterns of FPF, FUIO66 and simulated UIO66. a–d) Reproduced with permission [137]. Copyright 2025 Elsevier. e) LLTO solid electrolytes derived from 2D MOF lamellar membranes. Reproduced with permission [139]. Copyright 2022, Elsevier.

The preparation strategies for 2D MOF composite electrolytes can be informed by established methods for their 3D counterparts. Conventional 3D MOF composite solid electrolytes typically involve constructing continuous 3D networks, for instance, by in situ growth of ZIF‐67 nanoparticles on a cellulose framework (ZIF‐67@CF) [100] or by coordinating KOH‐etched ZIF‐8 nanocrystals onto a polyimide fiber nonwoven surface (Figure 7a–c) [101]. An alternative approach involves chemical modification of MOFs, such as synthesizing fluorine‐functionalized F‐UIO‐66 for the composite electrolyte FPF@PDOL (Figure 8a–d) [137] or preparing ZIFs grafted with amino and sulfonic acid groups (NH_3_^+^ SO_3_^−^@ZIF) [138]. These functionalized MOFs are then integrated with polymer matrices, lithium salts, ionic liquids, or other fillers to form 3D MOF composite solid electrolytes.

These methodologies provide a foundational framework for developing 2D MOF composite electrolytes, where 2D MOFs can be incorporated into matrices using similar dispersion, assembly, or in situ growth techniques. The resulting composites not only retain the intrinsic properties of the pristine 2D MOFs but also exhibit synergistic effects from the constituent materials. These synergies collectively enhance interfacial compatibility, mechanical stability, and surface functionality, thereby improving overall electrolyte performance.

Following a synthesis approach analogous to that used for 3D MOF composites, the most commonly employed method is the solution casting technique. In this process, 2D MOFs, polymer matrix, and lithium salt are dissolved in a solvent under overnight stirring. The resulting solution is then cast onto a polytetrafluoroethylene (PTFE) mold and finally dried under vacuum to obtain the 2D MOF composite polymer electrolyte [99]. Alternatively, 2D MOF composites can also be fabricated using an in‐situ induced growth strategy. For example, by impregnating inorganic LLTO precursors (LiNO_3_, La (NO_3_)_3_ 6H_2_O, Ti (OC_4_H_9_)_4_, etc.) into the pores and interlayer channels of 2D MOFs layered membranes, and then sintering them in situ at 1000°C, 2D MOFs layered membranes derived from LLTO electrolytes can be obtained as MLLTO, as shown in Figure 8e. The composite MLLTO has a lamellar structure; and the confinement effect of the MOFs interlayer channels regulates the orderly arrangement of the LLTO crystals along the c‐axis; meanwhile, the porous structure of the MOFs nanosheets enables the formation of a “pillar” structure between the layers and the construction of a vertical Li^+^ transport pathway [139]. Synthesis can be achieved through other specific methods. For instance, a “lithiation‐modified and solvent‐confined assembly” strategy is employed to prepare MOF‐X@DMF(Li) SSE. In this process, pristine MOF nanosheets are first lithiated by stirring with a 1 M lithium perchlorate (LiClO_4_) solution in DMF under oil‐bath heating. Subsequently, the lithiated nanosheets are stacked into a lamellar framework via low‐pressure vacuum filtration, followed by a heating step to allow for DMF volatilization and re‐infiltration, which promotes thorough solvent‐mediated assembly. This procedure ultimately yields a dense and defect‐free 2D MOF composite solid‐state electrolyte [84].

In summary, 2D MOF composites demonstrate unique structural advantages achieved through layered crystal design, hierarchical pore construction, interfacial synergy, and functional integration. Their performance is governed not only by the inherent designability of the MOF component but also by the synergistic effects arising from the combination with polymer matrices and inorganic materials

## 4 Ion Transport Mechanism and Performance Optimization

### 4.1 Ion Migration Pathways

Lithium ion transport in 2D MOF‐based SSEs occurs through several primary pathways:

#### 4.1.1 Interfacial Hopping

**FIGURE 9:**
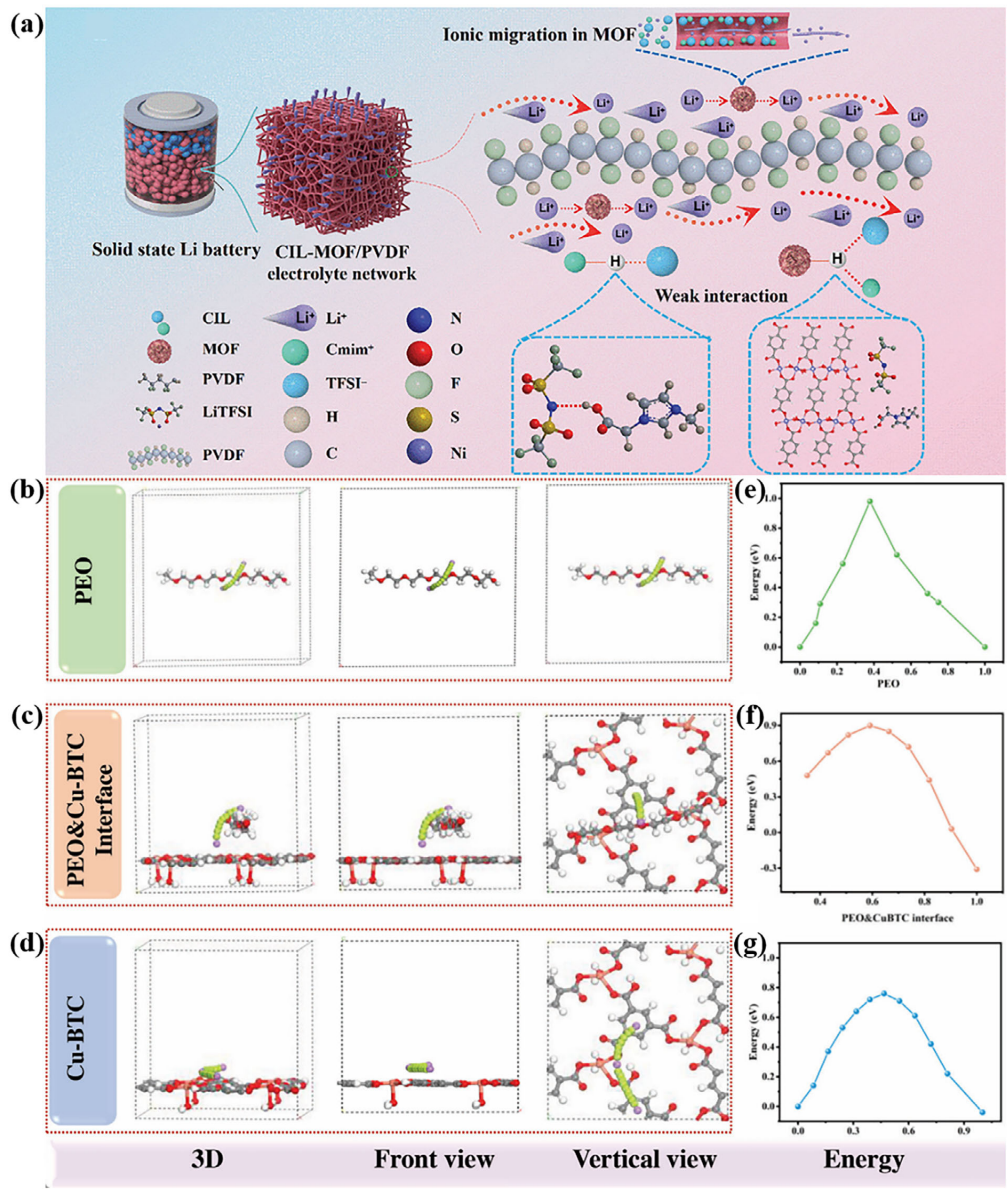
a) Schematic illustration of the battery architecture and Li^+^ hopping transport at PVDF/MOF interfaces. Reproduced with permission [140]. Copyright 2025, Wiley‐VCH. Migration mechanism of Li^+^ b) in PEO, c) on PEO/Cu‐BTC interface and d) on Cu‐BTC based on DFT. Migration energy barrier of Li^+^ e) in PEO, f) on PEO/Cu‐BTC interface and g) on Cu‐BTC based on MD simulation. b‐g) Reproduced with permission [81]. Copyright 2023, Wiley‐VCH.

Interfacial hopping serves as a key mechanism for ion migration, occurring at multiple active sites including pore edges within 2D MOF layers, centers of in‐plane benzene rings, grain boundaries (both intralayer and interlayer), and interfaces with other components. In 2D MOF‐based composite solid‐state electrolytes, the most prevalent pathway involves Li^+^ migration along polymer electrolyte matrix chains, a phenomenon observed across most MOF‐polymer systems. Within these systems, ions can hop between MOF layer surfaces and adjacent polymer chains. The inherent flexibility of polymer segments and their polar functional groups—such as ether oxygen atoms in PEO—enables them to effectively “bridge” adjacent MOF layers, forming continuous ion transport networks. In this process, ions initially adsorb onto MOF surfaces, subsequently hop onto polymer chains, migrate along these chains, and eventually transfer to neighboring MOF layers or other polymer domains (Figure 9a) [140].

However, this discrete hopping mechanism is highly dependent on local rapid segmental motion and conformational relaxation of polymer chains. Considering the inherent crystallization tendency of common polymer matrices like PEO and PVDF, strategic regulation of 2D MOF nanostructures—including pore size, specific surface area, and crystal morphology—can effectively suppress crystallization. For instance, the incorporation of high‐aspect‐ratio nanostructures inhibits regular folding of PEO chains, significantly reducing crystallinity while increasing the proportion of amorphous regions. This structural modification enables efficient Li^+^ migration through both MOF channels and polymer amorphous regions, even at low temperatures [79]. A representative example involves unsaturated Ni atoms on nickel‐based 2D MOF nanosheets (NMS), which bind with TFSI^−^ anions in LiTFSI through Lewis acid‐base interactions, thereby promoting lithium salt dissociation and liberating more free Li^+^ [141].

Beyond polymer‐mediated hopping, Li^+^ can also migrate rapidly along benzene ring centers within the 2D planes of Cu‐BTC MOFs. The electronegative centers of benzene rings provide continuous low‐energy migration sites, with calculated energy barriers significantly lower than those along PEO matrices (Figure 9b–d) [81].

#### 4.1.2 Interlayer Nanochannels

**FIGURE 10:**
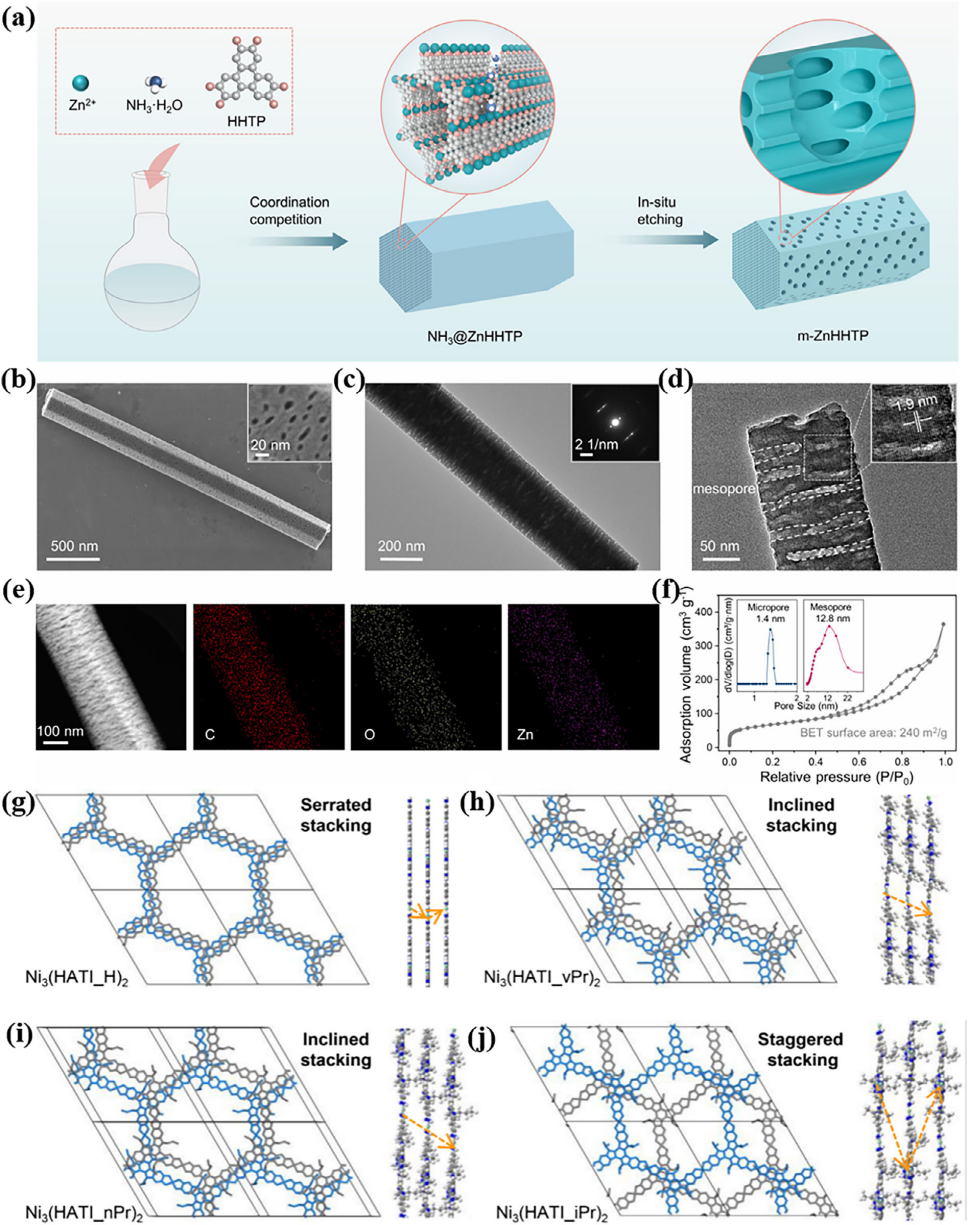
a) Schematic illustration of the synthesis for m‐ZnHHTP. b) FE‐SEM, c) TEM and SAED pattern, d) HRTEM image, e) EDS elemental mapping, and f) nitrogen adsorption‐desorption isotherms of m‐ZnHHTP. The insets in f) show micropore and mesopore size distribution curves. a–f) Reproduced with permission [142]. Copyright 2025 Wiley‐VCH. Top and side views of the corresponding fine 2D crystal structures of g) Ni_3_(HATI_H)_2_, h) Ni_3_(HATI_vPr)_2_, i) Ni_3_(HATI_nPr)_2_ and j) Ni_3_(HATI_iPr)_2_, respectively. g–j) Reproduced with permission [143]. Copyright 2024, American Chemical Society.

Interlayer nanochannels serve as the primary pathways for ion migration, occurring within the interlayer spacing of adjacent 2D MOF nanosheets. These slit‐like 2D nanochannels, formed by the stacking of layers, provide continuous and efficient conduits for ion transport. This structural feature represents the most distinctive ion migration mechanism of 2D MOFs compared to their 3D counterparts. Through tandem assembly‐etching chemistry, vertically aligned mesopores can be engineered within the microporous framework, resulting in a hierarchical “microporous–mesoporous” architecture. The mesopores establish lateral transport pathways that enable ions to traverse multiple microporous layers directly, thereby accelerating cross‐plane diffusion (Figure 10a–f) [142]. Structural analyses (Figure 10g–j) demonstrate that the incorporation of bulky substituents alters the stacking mode of the 2D MOF material Ni_3_(HATI_iPr)_2_ from a herringbone to a staggered arrangement, leading to an expansion of the interlayer distance. This interlayer offset and increased spacing weaken π‐π interactions, resulting in more open pore structures that facilitate in‐plane ion diffusion [143]. For example, layered NiCo‐MOF self‐assembles into interlayer nanochannels with pore sizes of 1.0–2.0 nm, which are filled with succinonitrile (SN) electrolyte. Within these channels, SN molecules align horizontally under the coordination guidance between Co/Ni metal sites and C≡N groups, forming directional pathways for Li^+^ migration [144]. Furthermore, functionalization with ‐NH_2_ groups enhances the electron‐donating character of the 2D MOF, promoting anion adsorption and restricting anion mobility, thereby compelling Li^+^ ions to preferentially migrate through the interlayer channels [83]. The interlayer distance is a critical parameter governing ion transport efficiency. Effective ion conduction requires that this spacing be expanded to dimensions comparable to or larger than the diameter of the target ion. This interlayer spacing can be dynamically modulated through strategies such as intercalation, external stimuli (e.g., pressure, temperature, or electric fields), or chemical modification (e.g., grafting of bulky functional groups), enabling optimization of the nanochannel dimensions for enhanced ion conduction.

#### 4.1.3 Internal Channels of 2D MOFs

Internal channels of 2D MOFs serve as another primary pathway for ion transport, occurring within individual nanosheets through continuous, highly ordered in‐plane pore architectures. These structures typically consist of transition metal ions coordinated with organic ligands to form hexagonal honeycomb frameworks. The uniformly arranged, dimensionally consistent in‐plane nanochannels establish structurally selective pathways for ion migration, while open metal sites (OMS) further enhance conduction efficiency by anchoring anions and liberating mobile charge carriers.

**FIGURE 11:**
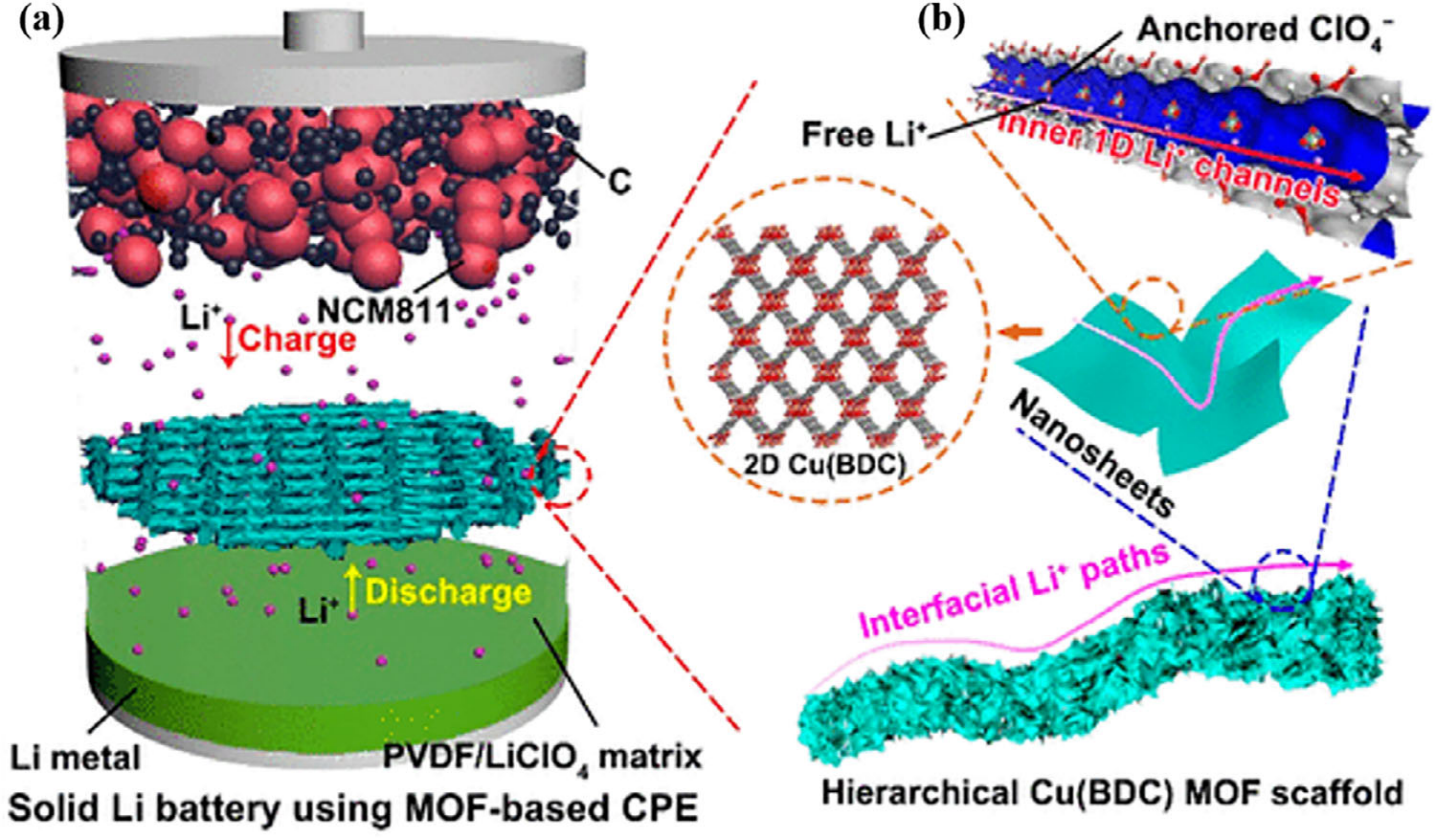
a) Schematic of the CPE design for Cu(BDC). b) Schematic diagram of Li^+^ intra‐layer transport path along the Cu(BDC) MOF aperture. a,b) Reproduced with permission [82]. Copyright 2021, American Chemical Society.

When cobalt‐based MOFs nanosheets (CMS) are incorporated into PVDF matrices, Li^+^ migration occurs not only through amorphous PVDF domains but also through CMS's porous topology featuring 1.85 nm pores, collectively enhancing transport efficiency [77]. Similarly, Cu(BDC) possesses ordered 1D nanochannels with approximately 0.67 nm diameter, where pore dimensions closely match Li^+^ ionic radii. The OMS in these channels preferentially adsorb ClO_4_^−^ anions, releasing free Li^+^ for directional migration along these low‐resistance pathways (Figure 11) [82].

The intrachannel transport pathways, formed by coordination bonding between metal nodes and organic ligands, require optimal dimensions and high interconnectivity to enable efficient ion migration. Undersized diameters or discontinuous pathways severely impede ionic mobility. Precise tuning of channel size and chemical environment—achieved through ligand structural engineering, metal node selection, or advanced assembly strategies—enables accommodation of diverse ionic transport requirements.

#### 4.1.4 Synergistic Transport Mechanisms

**FIGURE 12:**
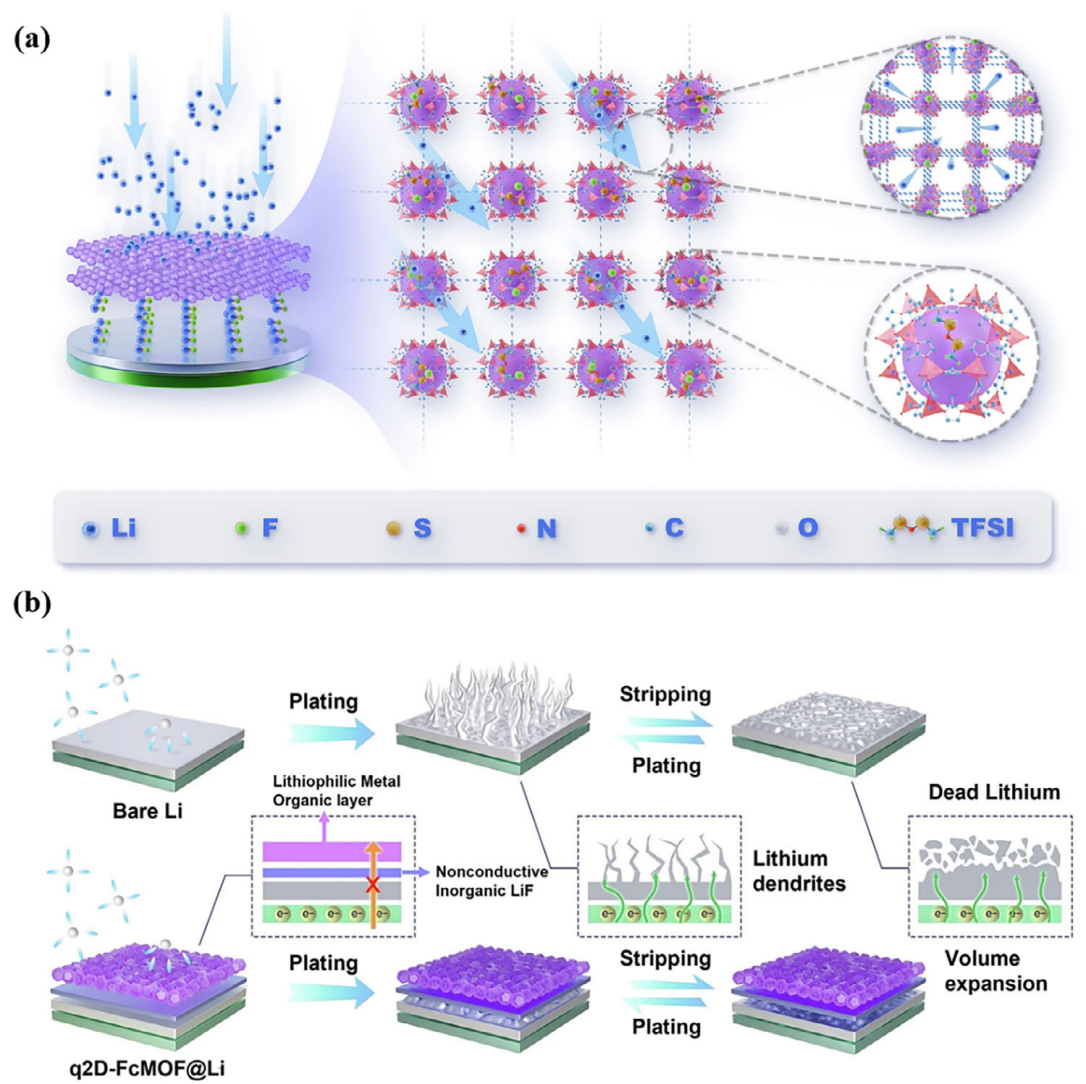
Schematic illustration of microscopic strategy to manipulate incorporating organometallic frame/inorganic LiF hybrid double‐layer ASEI. a) ASEI formed on Li with immobilized anions and ionic channels for fast Li^+^ transport. b) Li deposition behaviors on bare Li and q2D‐FcMOF@Li. a,b) Reproduced under the terms of the CC‐BY‐NC‐ND license [145]. Copyright 2025, Springer Nature.

Synergistic transport mechanisms represent advanced ion conduction pathways that emerge from strategic modifications of solid‐state electrolytes or their composites with inorganic materials. A notable example is the fluorinated q2D‐FcMOF, which forms an artificial solid electrolyte interphase (ASEI) bilayer with distinct Li^+^ migration behaviors. In the outer metal‐organic framework layer, Li^+^ accumulates around high‐adsorption‐energy metal sites, creating localized high‐concentration zones that reduce nucleation overpotential, while the ordered nanochannels provide low‐tortuosity pathways that prevent local ion depletion. Within the inner LiF inorganic layer, structural defects act synergistically to facilitate Li^+^ hopping migration, leveraging the intrinsic low diffusion barriers and high surface energy of LiF to enable rapid ion conduction while maintaining electronic insulation (Figure 12) [145].

In another configuration, the composite of 2D MOFs with LLTO enables predominant lattice‐channel migration. The layered precursor structure derived from the MOF template promotes continuous alignment of LLTO grains, forming interconnected Li^+^ highways that minimize grain‐boundary resistance and significantly enhance ion mobility [139].

### 4.2 Performance Optimization Strategies

#### 4.2.1 Chemical Modification

Chemical modification serves as a powerful strategy for precisely regulating interfacial properties and ion transport behavior in solid‐state electrolytes through the introduction of functional groups such as ‐SO_3_H, ‐NH_2_, and others. This approach not only enhances selective adsorption and dissociation of lithium salts, providing abundant active sites for ion conduction, but also optimizes interlayer electric field distribution through charge interactions, thereby reducing ion migration barriers.

From the perspective of interaction with lithium, the ‐SO_3_H group exhibits strong polarity and negative charge. Upon dissociation, the resulting sulfonate (‐SO_3_^−^) ion forms stable coordination with Li^+^ through electrostatic forces. This mechanism promotes the dissociation of lithium salts to release more free Li^+^ ions while simultaneously anchoring the anions to restrict their migration. This reduces transport resistance caused by ion agglomeration, thereby enhancing Li^+^ migration [146]. Simultaneously, the spatial site‐blocking effect of functional groups enables precise regulation of interlayer spacing and pore architecture, balancing structural order with pathway flexibility to prevent mass transfer limitations caused by excessive layer stacking. The introduction of amino (‐NH_2_) or nitro (‐NO_2_) groups substantially increases MOF porosity, enhancing interfacial contact area with polymer electrolytes and expanding ion transport pathways. The ‐NH_2_ serves as a typical electron‐donating group, whose nitrogen atom's lone pair electrons can form weak coordination with Li^+^. This not only lowers the migration energy barrier for Li^+^ within the MOF channels but also optimizes the electric field distribution through charge polarization effects, guiding Li^+^ transport along low‐resistance pathways [83]. From the perspective of structural stability, both types of functional groups exhibit strong polarity. The hydroxyl group of ‐SO_3_H can form hydrogen bonds with the ligands within the MOF layers, while the nitrogen atom of ‐NH_2_ can engage in weak coordination interactions with the metal nodes. Both mechanisms enhance interlayer interactions or framework density without disrupting the strong intralayer coordination bonds or the π‐π conjugation network, thereby preventing structural collapse that might otherwise result from the introduction of these functional groups.

Furthermore, fluorination of metal nodes in 2D MOFs enables the formation of metal fluorides, while fluorine atoms embedded within the carbon framework create an inorganic LiF layer characterized by high ionic conductivity and electronic insulation. This dual functionality accelerates Li^+^ conduction while blocking electron penetration, effectively suppressing side reactions [145]. By combining diverse functional group modifications with rational structural design, chemical tailoring of solid‐state electrolytes opens new avenues for interface engineering in high‐performance lithium‐metal batteries.

#### 4.2.2 Interlayer Engineering

Interlayer engineering through intercalation—such as with ionic liquids—enables precise regulation of interlayer spacing and the local ion‐transport environment. Leveraging the molecular dimensions and polarity of the intercalant, species can be embedded between adjacent layers, physically expanding the interlayer distance. This expansion creates wider transport channels for ion migration, effectively reducing mass‐transfer resistance induced by layer stacking.

When ionic liquids are used as intercalants, their high ionic conductivity and low volatility further contribute to the formation of a lubricating interlayer phase. This not only enlarges the interlayer spacing but also significantly lowers the energy barrier for ion migration [147]. Moreover, the intercalation process allows dynamic modulation of interlayer interactions, enhancing structural flexibility while preserving framework rigidity. Such adaptability accommodates volume changes during battery cycling, offering an effective strategy—based on spatial interlayer regulation—to optimize ionic conduction efficiency and interfacial compatibility in solid‐state electrolytes. Consequently, the insertion of ionic liquids tailored to MOF pore dimensions provides valuable design insight for the application of 2D MOFs in advanced solid‐state electrolyte systems

#### 4.2.3 Composite Design

**FIGURE 13:**
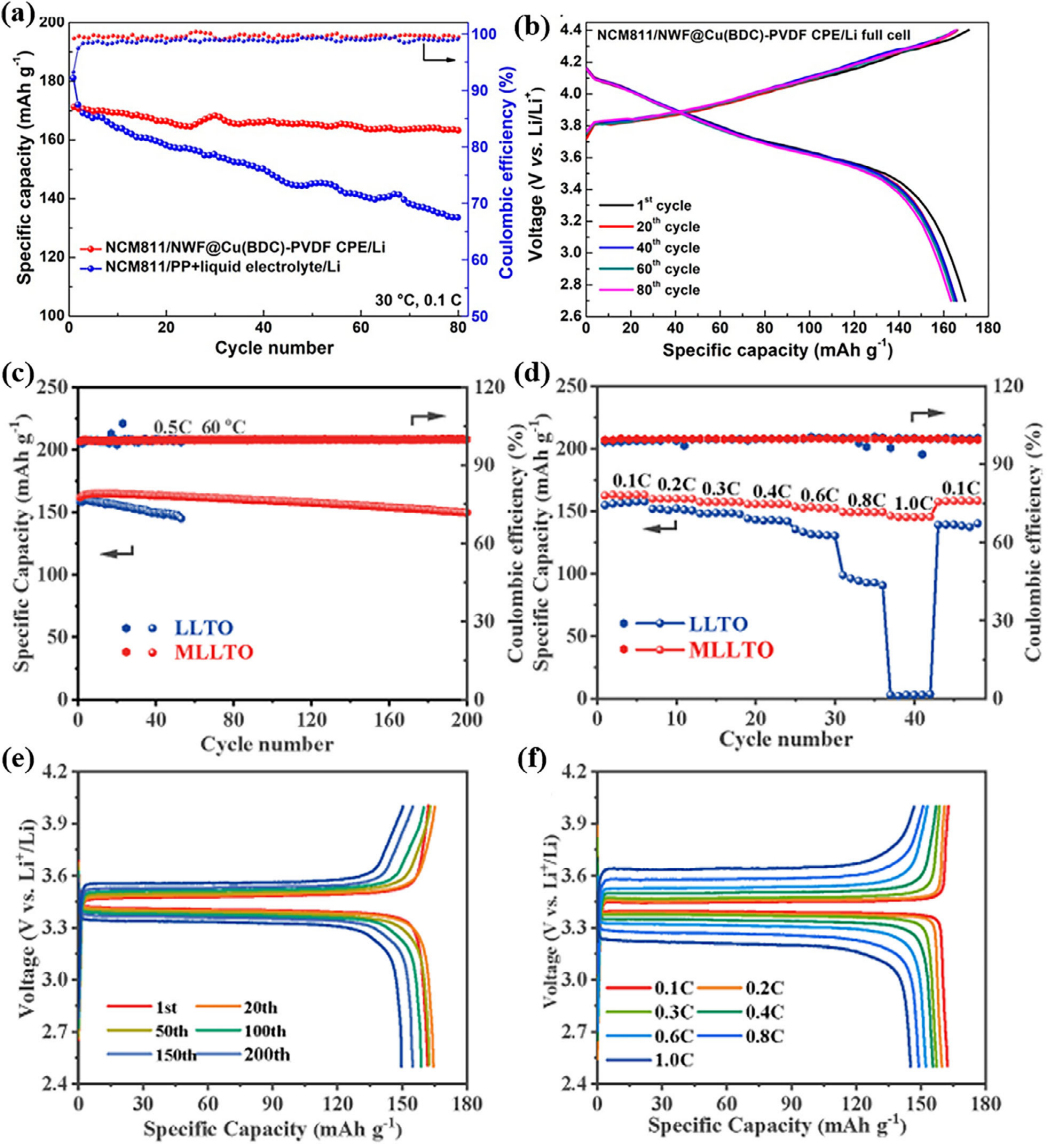
a) Cycling performance and b) voltage profile of NCM 811/NWF@Cu (BDC)‐PVDF CPE/Li cell at 0.1 C 30°C. a,b) Reproduced with permission [82]. Copyright 2021, American Chemical Society. c) Cycling performance of LFP/MLLTO/Li and LFP/LLTO/Li cells at 0.5 C 60°C. d) Performance of LFP/MLLTO/Li and LFP/LLTO/Li cells at different multiplications. e) Voltage profile of LFP/MLLTO/Li cell at 0.5 C 60°C. f) Voltage curves of LFP/MLLTO/Li cells at different multiplications. c–f) Reproduced with permission [139]. Copyright 2022, Elsevier.

Composite design using 2D MOFs enables synergistic optimization of electrolyte properties through strategic integration with various functional materials. For instance, in situ growth of 2D MOF nanosheets on nonwoven fabric (NWF) substrates forms an interconnected 3D network architecture characterized by enhanced specific surface area and improved hydrophilicity. This configuration significantly promotes uniform electrolyte infiltration and expands the interfacial contact area between MOFs and the electrolyte matrix. The interconnected nanosheet network combined with strong adhesion to the NWF substantially enhances the mechanical robustness of the composite electrolyte, effectively resisting lithium dendrite penetration and ensuring long‐term cycling stability [82], as shown in Figure 13a,b.

Alternatively, a novel composite electrolyte MLLTO can be constructed by integrating 2D MOFs with the inorganic solid‐state electrolyte LLTO. In this system, the confined interlayer channels of the 2D MOFs guide the oriented growth of LLTO crystals, minimizing grain boundary defects and resistance while reducing interfacial impedance. Simultaneously, the orderly arrangement of LLTO crystals within the interlayer spaces establishes continuous ion conduction pathways, achieving dual enhancement of ionic conductivity and electrochemical stability while maintaining excellent mechanical strength [139]. The corresponding battery performance is demonstrated in Figure 13c–f.

The composite strategy of 2D MOFs with either polymeric or inorganic materials creates interfacial synergistic effects that enhance mechanical integrity and interfacial compatibility, forming multi‐channel ion transport networks. By simultaneously regulating framework defects and surface chemistry, this approach optimizes ion migration kinetics while suppressing lithium dendrite growth. Through structural complementarity and functional integration, this composite design strategy achieves synergistic improvements in ionic conductivity, electrochemical stability, and mechanical properties of solid‐state electrolytes.

## 5 Typical Material Systems and Applications

### 5.1 BDC (Terephthalic Acid) and Derivatives as a Classical Ligand for 2D MOFs in Solid‐State Electrolytes

BDC (terephthalic acid, C_8_H_6_O_4_) and derivatives serves as a classical carboxylic acid ligand for constructing 2D MOFs and represents the most widely used synthetic ligand for solid‐state electrolyte applications. Its molecular structure contains two or three carboxylic acid groups (‐COOH) symmetrically positioned at out sites of the benzene ring, forming a linear and planar configuration. The rigid conjugated benzene ring backbone, combined with the bi‐ or poly‐dentate coordination capability of the carboxylate groups, enables the formation of stable metal‐oxygen bonds with metal nodes, facilitating the assembly of well‐defined two‐dimensional layered networks

The rigidity of BDC imparts regular porosity to the resulting MOFs, while the coordination behavior of the carboxyl groups can be modulated by reaction parameters such as pH and solvent, thereby influencing framework crystallinity and stability. A representative 2D MOF, Cu(BDC), features a structural unit comprising two five‐coordinated Cu^2+^ ions bridged in a paddle‐wheel arrangement. Each Cu^2+^ ion coordinates with two BDC^2−^ ligands in a bidentate bridging mode, while also binding a terminal DMF solvent molecule to form a [Cu(BDC)(DMF)] coordination unit. This structure crystallizes in a monoclinic system with inherent anisotropy, favoring 2D layered growth [148].

Stacking of these two‐dimensional layers generates ordered 1D pore channels, which provide fast transport pathways for Li^+^ and offer a high specific surface area conducive to enhanced interfacial interaction with polymer electrolytes. Additionally, each Cu^2+^ site contains an accessible uncoordinated metal site capable of selectively adsorbing anions, thereby liberating free Li^+^ and promoting ionic conduction [82]. As a typical 2D MOF, Cu(BDC) achieves high ionic conductivity, robust mechanical properties, and a wide electrochemical window through the synergistic combination of its layered nanosheet morphology, open metal sites, and continuous porous network. This offers a molecular‐level engineering strategy for designing high‐performance composite polymer electrolytes.

### 5.2 BHT as a Polydentate Ligand for 2D MOFs

BHT (hexamercaptobenzene, C_6_H_6_S_6_) is a polydentate ligand capable of forming metal‐sulfur (M‐S) bonds through coordination between its six thiol groups and metal ions or clusters such as Zn^2+^, Cu^2+^, and Ni^2+^. The benzene ring serves as a rigid planar scaffold, while the thiol groups function as bridging units that link metal centers into an extended two‐dimensional network via S‐metal coordination.

The resulting 2D MOF layers are stabilized by strong coordination bonds within the plane and stack through π‐π interactions or weak van der Waals forces, leading to materials with high specific surface area and tunable porosity. A representative example, Cu‐BHT, consists of Cu^2+^ ions coordinated with C_6_H_6_S_6_ ligands via Cu‐S bonds, forming a planar 2D structure. It crystallizes in a highly symmetric hexagonal system belonging to the D_6h_ point group and exhibits a Kagome‐type lattice, in which the dense arrangement of ligands and metal ions creates high‐density adsorption sites [149].

The sulfur‐rich heterocyclic structure of BHT‐based 2D MOFs provides numerous potential lithium‐ion binding and transport sites. Combined with their intrinsic hydrophobicity, these materials show promising potential for application in solid‐state electrolytes.

### 5.3 HIB as a Nitrogen‐Rich Ligand for 2D Conductive MOFs

HIB (hexa‐iminobenzene, C_6_(NH)_6_) features six imino (‐NH‐) groups uniformly arranged on a benzene ring, forming a rigid planar structure with six‐fold axial symmetry. Each nitrogen atom in the imino group serves as a coordination site, enabling the formation of a hexagonal lattice with transition metal ions such as Cu^2+^, Ni^2+^, and Pt^2+^, thereby constructing a honeycomb‐type 2D layered framework.

The nitrogen‐rich heterocyclic ligand acts as a strong Lewis base, which significantly promotes lithium salt dissociation and facilitates Li^+^ transport. The planar architecture of HIB enables stable interlayer stacking through π‐π interactions and N‐H hydrogen bonding, allowing precise regulation of interlayer spacing and porosity.

A representative material, Co‐HIB, is formed by coordinating amine‐functionalized organic ligands with cobalt ions. In this structure, the cobalt centers and HIB aromatic frameworks are surrounded by hydrogen atoms, creating a hydrogen‐rich environment within the MOF pores. This enhances hydrogen‐bonding interactions and facilitates the formation of hydrogen‐bonding networks with polymer electrolytes such as PEO, effectively suppressing crystallization and improving mechanical strength.

Moreover, Co‐HIB exhibits a high cobalt density of 7.69% [150], providing abundant Lewis acid metal sites. These sites strongly anchor anions such as TFSI^−^ and FSI^−^, weakening ion‐pairing interactions and enhancing lithium‐ion mobility.

### 5.4 2D MOF Composite Electrolytes

2D MOF composite electrolytes represent a class of solid‐state electrolyte systems formed by integrating two‐dimensional metal‐organic frameworks with polymer matrices, ionic liquids, or inorganic fillers. Their defining characteristic lies in the unique layered topology of 2D MOFs, which consist of atomically thin metal–organic sheets stacked into ordered interlayer ion channels. These structures offer high specific surface area and chemically tunable pore environments, while the incorporation of secondary materials further synergistically enhances the overall electrolyte performance.

**FIGURE 14:**
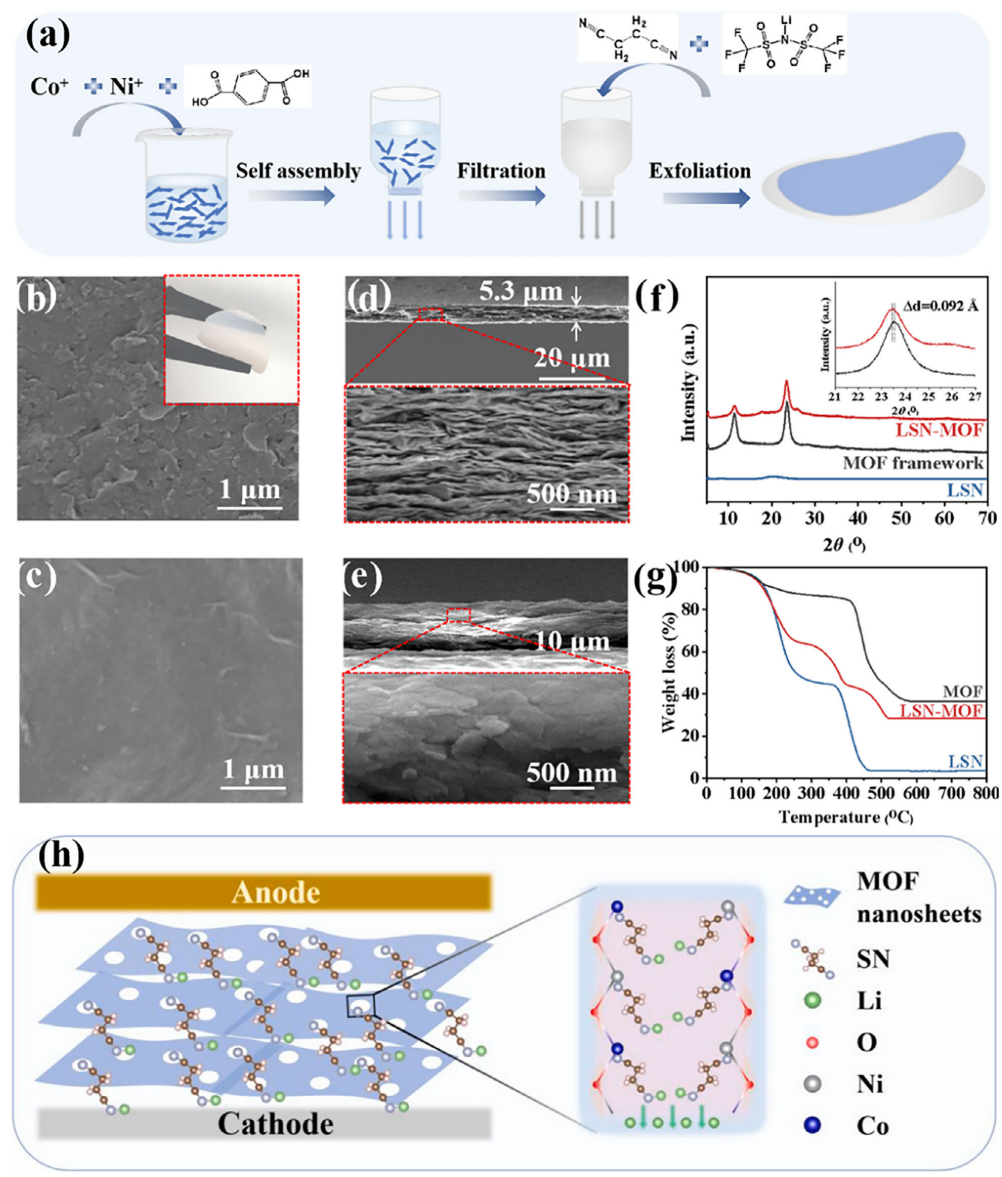
a) Schematic preparation process of LSN‐MOF CSE. Surface SEM images of the b) laminar MOF framework and c) LSN‐MOF CSE. Cross‐sectional SEM images of d) laminar MOF framework and e) LSN‐MOF CSE. f) XRD patterns and g) TG curves of laminar MOF framework, LSN electrolyte and LSN‐MOF CSE. h) Schematic of the CPE design of the LSN‐MOF composite electrolyte and the SN arrangement and Li^+^ transport in the LSN‐MOF nanopores. a‐h) Reproduced with permission [144]. Copyright 2023, Elsevier.

For instance, a composite solid electrolyte (CSE) was fabricated by infiltrating a LiTFSI‐succinonitrile (LSN) electrolyte into the interlayer nanochannels of a 2D MOF. In this LSN‐MOF system, the LSN medium provides ample storage space while preserving the mobility of SN molecules. Simultaneously, unsaturated metal sites within the 2D MOF coordinate with the C≡N groups of SN, inducing horizontal alignment of the SN molecules within the nanopores and facilitating directional vertical transport of lithium ions [144], as illustrated in Figure 14.

The electrochemical properties of several currently studied 2D MOF‐based solid‐state electrolytes are shown in Table 3. We can optimize performance by regulating the structure of 2D MOFs. Layered or vertically aligned 2D MOFs enhance transport efficiency by shortening ion transport pathways. For instance, the ionic conductivity of vertically extended Cu‐HHTC and layered Cu(BDC‐2OH) is significantly higher than that of randomly stacked MOFs [84, 99, 141]. Furthermore, the benzene ring of BDC can be functionally modified through amination, halogenation, or incorporation of bimetallic sites to introduce additional functionalities. The ‐OH containing Cu(BDC‐2OH) induces DMF to form a low‐energy‐barrier transport layer via hydrogen bonding [99]. The ‐NH_2_ functionalized CoFe‐BDC‐NH_2_ restricts anion migration through an electron‐donating effect [83]. The fluorinated q2D‐FcZ8 can form a LiF‐rich SEI layer [145]. Concurrently, interfacial adaptation plays a crucial role. Interfacial interactions between MOFs and electrolytes/electrodes determine cycling stability. For instance, Cu‐HHTC induces π‐d electron delocalization to form LiF‐rich SEI, while q2D‐FcZ8 constructs a dual‐layer ASEI enabling symmetric cell cycling lifetimes exceeding 3200–3600 h [84, 145]. Based on this, we can design layered/vertically aligned crystal structures to construct continuous transport pathways. Functional group modifications optimize the ionic solvation environment and lithium salt dissociation efficiency. Simultaneously, leveraging the specific interactions between MOFs and ions enables the construction of stable interfacial layers, achieving synergistic high ionic conductivity and long‐term cycling stability.

**TABLE 3:** Electrochemical properties of 2D MOF‐based solid‐state electrolytes.

2D MOFs type	Metal center	Ligand	Ionic conductivity @RT (mS cm^−1^)	$t_{Li^{+}}$	Current density, (mA cm^−2^) cycle life (h)	Cathodes	Capacity after cycling (mA h g^−1^)	Refs.
Cu‐BTC	Cu^2+^	Homophthalic acid (BTC)	0.046	0.58	0.1, 1300 at 60°C	LiFePO_4_	162 (500th cycle at 0.5C)	[81]
CuBDC‐10	Cu^2+^	Terephthalic acid (BDC)	1.75	0.77	0.2, 1800	LiFePO_4_	130 (50th cycle at 0.1C)	[79]
q2D‐FcZ8	Zn^2+^	2‐methylimidazole (fluorinated)	—	0.52	1.0, 3600	LiFePO_4_	133 (600th cycle at 1C)	[145]
NMS	Ni^2+^	Terephthalic acid (BDC)	0.0166	0.378	0.3, 900 at 50°C	LiFePO_4_	130 (50th cycle at 0.1C)	[141]
CMS	Co^2+^	Terephthalic acid (BDC)	0.626	0.70	0.1, 750	LiFePO_4_	140 (650th cycle at 0.5C)	[77]
Cu (BDC)	Cu^2+^	Terephthalic acid (BDC)	0.24	0.61	0.2, 300	NCM811	164 (80th cycle at 0.1C)	[82]
NiCo‐MOF	Ni^2+^ Co^2+^	Terephthalic acid (BDC)	0.741	0.59	0.2, 800 at 25°C	LiFePO_4_	148 (200th cycle at 0.2C)	[144]
NiCoMLLTO	Ni^2+^ Co^2+^	Terephthalic acid (BDC)	0.119	—	0.2–0.4, 1000 at 60°C	LiFePO_4_	149.6 (200th cycle at 0.5C)	[139]
CoFe‐BDC‐NH_2_	Co^2+^ Fe^3+^	Terephthalic acid (BDC)	0.065	0.64	0.2, 500 at 60°C	NCM523	138.1 (100th cycle at 0.2C)	[83]
Cu(BDC‐2OH)	Cu^2+^	2,5‐Dihydroxyterephthalic acid (BDC‐2OH)	0.776	0.81	0.2, 1000 at 0°C	LiFePO_4_	171.2 (300th cycle at 0.5C)	[99]
Cu‐HHTC	Cu^2+^	2,3,6,7,14,15‐Hexahydroxyltriptycene (HHTC)	1.59	0.81	0.1, 3200	LiFePO_4_	115.4 (1000th cycle at 1C)	[84]

## 6 Challenges and Outlook

### 6.1 Key Challenges

#### 6.1.1 Scale‐up Preparation: Difficulties in Achieving Large‐Area Homogeneity

The primary obstacle to the large‐area homogeneous preparation of 2D MOFs lies in the inherent mismatch between nucleation and growth kinetics. Firstly, non‐uniform nucleation density presents a major issue. In solution‐based methods, local concentration gradients of metal ions and ligands—often caused by factors such as temperature variations within the reactor during solvothermal synthesis—lead to a discrete distribution of nucleation sites and the formation of discontinuous “island‐like” films rather than continuous uniform layers.

Secondly, interlayer growth mismatch further complicates scalable production. The weak van der Waals forces that bond adjacent 2D MOF layers provide insufficient interlayer stability, making the structure prone to layer misalignment and slippage during large‐scale fabrication processes. Additionally, the strong interlayer interactions within 2D MOFs make their nanocrystals difficult to completely disperse in solution, fundamentally limiting the ability to produce uniform, ultrathin large‐area films through solution‐processing techniques [133].

#### 6.1.2 Interfacial Challenges

Interfacial issues between rigid 2D MOFs and transition metal electrode materials contribute to elevated interfacial impedance and undermine efforts to suppress lithium dendrite growth. These challenges can be fundamentally categorized into two aspects: physical mismatch and chemical side reactions.

Physical mismatch arises from disparities in the thermomechanical properties of the constituent materials. Differences in the coefficients of thermal expansion between 2D MOFs and transition metal substrates can cause interfacial stress accumulation during temperature fluctuations in charge‐discharge cycles, potentially resulting in crack formation or delamination of the MOF layer. Furthermore, the mechanical incompatibility between inherently rigid 2D MOFs and ductile metal electrodes can generate microcracks during repeated cycling, leading to degraded mechanical integrity and disruption of continuous ion transport pathways [151].

Chemical side reactions primarily involve metal node corrosion and unstable solid electrolyte interphase (SEI) formation. Transition metals may undergo ligand exchange reactions with organic linkers in the MOF structure, generating resistive interfacial layers that increase impedance. Simultaneously, the interface between MOFs and metal electrodes tends to form inhomogeneous SEI films rich in high‐resistance components such as Li_2_CO_3_ and LiOH, which further impede efficient lithium‐ion transport.

#### 6.1.3 Chemical Stability

Although 2D MOFs exhibit structural stability, this stability is inherently conditional and remains effective only within the material's structural tolerance threshold. In practical applications, environmental conditions often exceed this threshold. For instance, excessively high voltage may induce structural decomposition, while elevated temperatures can weaken interlayer interactions or trigger ligand decomposition, ultimately leading to framework collapse [152].

Acidic or alkaline environments further threaten structural integrity through distinct dissolution mechanisms. Under acidic conditions, hydronium ions (H_3_O^+^) attack metal centers, protonating metal‐ligand bonds and eroding coordination stability [153]. In alkaline media, hydroxide ions (OH^−^) compete with organic ligands for metal coordination sites, often forming metal hydroxides that disrupt the framework architecture.

Most 2D MOFs maintain structural and functional stability only within narrowly defined conditions: neutral to mildly acidic environments, moderate temperatures, and absence of strong redox activity [154]. This limited stability window poses significant challenges for their integration into robust energy storage systems.

#### 6.1.4 Mechanistic Studies: Limitations in Real‐Time Ion Transport Characterization

A significant challenge in understanding 2D MOF‐based electrolytes is the scarcity of in situ characterization techniques capable of directly revealing ion transport processes under operating conditions. Although several analytical methods are available [155, 156, 157, 158, 159, 160, 161], current approaches remain limited in their ability to monitor ionic motion in real time within solid‐state batteries. Conventional techniques such as X‐ray diffraction (XRD) and scanning electron microscopy (SEM) typically provide only static structural or morphological information, making it difficult to track dynamic ion migration pathways and kinetics during battery operation. While recent advances have led to the development of in situ XRD and SEM platforms, these methods still lack the spatial and temporal resolution required to precisely resolve the complex ion transport mechanisms within hierarchically structured 2D MOFs.

Ion transport in 2D MOF‐based systems involves multiple coupled processes, including intra‐pore migration, interfacial hopping, and interaction with polymer matrices. Existing characterization tools struggle to comprehensively capture the dynamics of these processes. Although ssNMR techniques can probe local ionic environments and interactions [162, 163, 164], they face limitations in quantitatively distinguishing ions within different chemical environments in 2D MOFs or in determining critical kinetic parameters such as site‐specific migration rates and residence times.

Theoretical modeling and simulation represent essential tools for deepening the mechanistic understanding of 2D MOFs in battery systems [165, 166, 167]. However, constructing accurate and representative atomic‐scale models remains challenging due to the structural complexity of 2D MOFs, which feature diverse coordination geometries, organic‐inorganic interfaces, and ubiquitous defects and vacancies. Accurately incorporating these structural features and their collective influence on ion transport behavior remains a persistent hurdle in computational studies.

#### 6.1.5 Insufficient Ionic Conductivity

The ionic conductivity of most MOF‐based electrolytes remains considerably lower than that of conventional liquid electrolytes. While liquid systems enable nearly unrestricted ion migration through bulk fluid phases, 2D MOFs—despite their structural advantages—impose greater resistance due to their tortuous pore networks. Although pore dimensions and chemical environments can be tailored to favor ion diffusion, the inherently zigzag migration pathways through crystalline frameworks significantly increase ionic transport resistance. Furthermore, residual solvent molecules trapped within MOF pores after synthesis are often difficult to completely remove, further impeding ion mobility and contributing to reduced conductivity compared to liquid systems [122, 168, 169].

Ion transport kinetics in 2D MOFs are also intrinsically slower. Migration relies on continuous coordination and de‐coordination with the framework. While moderate host‐guest interactions can guide directional transport, excessively strong binding can cause prolonged ion residence at specific sites, thereby limiting macroscopic migration rates. In contrast, the weaker and more dynamic solvation environments in liquid electrolytes support faster ionic motion. In 2D MOF‐polymer composite systems, the complex interplay of Li^+^ with both the MOF framework and polymer chains often results in limited lithium‐ion mobility, restricting overall conductivity to levels still below those of liquid electrolytes.

**FIGURE 15:**
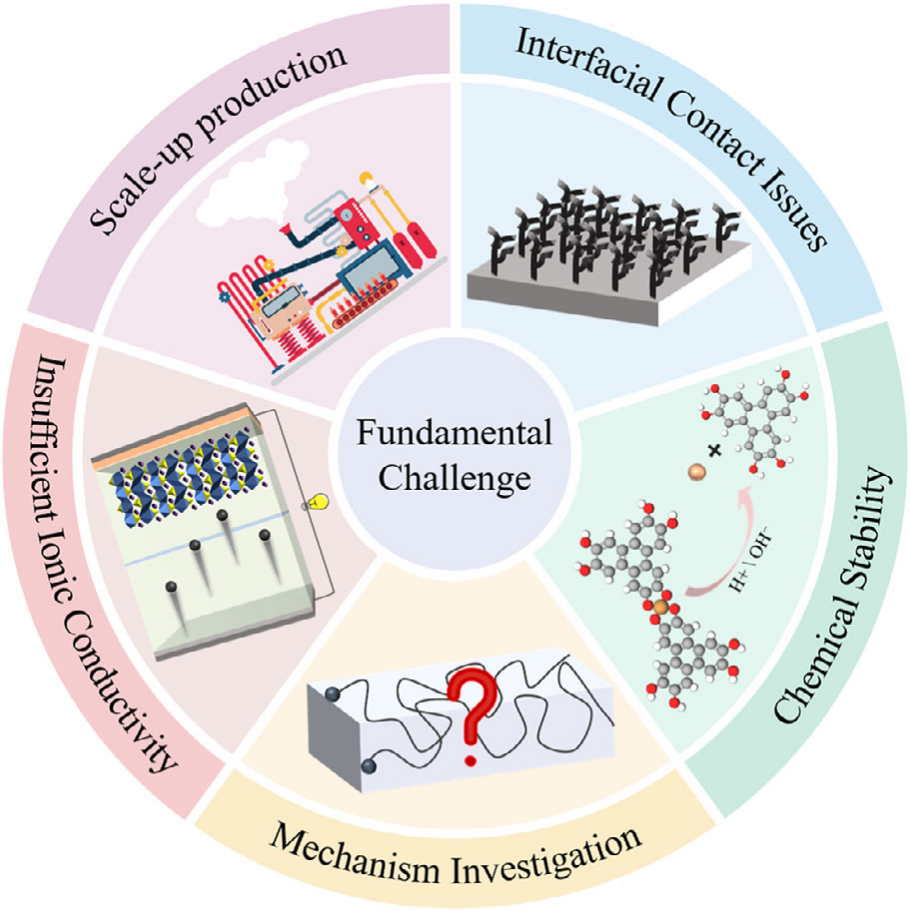
Key challenges for 2D MOF materials at this stage.

An additional challenge lies in enhancing conductivity without compromising stability. Certain strategies aimed at improving ion transport—such as introducing specific functional groups or guest molecules—may inadvertently weaken structural integrity. For instance, while guest intercalation can enhance interlayer spacing and facilitate conduction, the decomposition or desorption of these species under extreme temperatures can disrupt ion‐conducting pathways and degrade performance [170]. In contrast, liquid electrolytes generally maintain stable conduction across wider temperature and voltage ranges. Achieving a comparable balance between high ionic conductivity and structural stability remains a fundamental challenge for 2D MOFs, limiting their practical adoption (Figure 15).

### 6.2 Future Directions

#### 6.2.1 Development of Controlled Growth Technologies: Atomic Layer Deposition (ALD)

ALD is a vapor‐phase technique that enables precise, atomic‐level control over thin‐film growth [171, 172, 173]. In the synthesis of 2D MOFs, ALD offers the potential to achieve accurate regulation of structure and composition. By carefully selecting precursor types, tuning deposition cycles, and optimizing reaction conditions, ALD facilitates the layer‐by‐layer growth of 2D MOF films with tailored structures and functionalities on various substrates.

The ALD process relies on sequential, self‐limiting surface reactions, using pulse‐purge sequences to precisely control precursor exposure time and prevent gas‐phase reactions. A key consideration in MOF synthesis via ALD is balancing the reaction kinetics with the thermal stability of organic ligands. Several in situ and ex situ techniques support the characterization and optimization of such films: (1) In situ monitoring: Quartz crystal microbalance (QCM) or spectroscopic ellipsometry can track growth rates in real time. (2) Structural analysis: XRD confirms crystallinity, while X‐ray photoelectron spectroscopy (XPS) verifies elemental oxidation states. (3) Defect control: The density of grain boundaries—and thus charge carrier mobility—can be tuned by varying the number of ALD cycles.

This highly controlled synthesis strategy not only enhances the consistency and reproducibility of 2D MOF properties but also provides a versatile platform for developing new functional materials. Furthermore, layer‐by‐layer assembly techniques enable the conformal growth of 2D MOFs on ferromagnetic electrodes, where the thickness tunability, high crystallinity, and substrate adaptability of the resulting films offer a promising foundation for scalable production [174].

#### 6.2.2 Leveraging Structure‐Property Relationships in 2D MOFs for Designing High‐Performance Solid‐State Electrolytes

##### 6.2.2.1 Design of Advance Structures to Enhance Conductivity and Li^+^ Transference Number

2D MOFs with intrinsic ion channels represent an emerging class of materials featuring continuous and tunable ion transport pathways embedded within their native structures for ion conductivity. These channels originate from the inherent porosity and coordination chemistry of the MOFs, enabling efficient ion conduction without requiring external modification or composite formation. Such materials show significant potential for application in solid‐state electrolytes. Structural features of intrinsic ion channels for conductivity: (a) In‐plane Ordered Porosity: Periodic 1D or 2D pore arrays are formed within the 2D plane through coordination between metal nodes and organic ligands; (b) Interlayer nanochannels: Interlayer spacing stabilized by weak non‐covalent interactions (e.g., π‐π stacking, van der Waals forces) provides secondary pathways for ion migration; (c) Tailored chemical microenvironment: Functional groups such as ‐SO_3_H, ‐NH_2_, and ‐ COOH grafted on pore walls regulate ion selectivity and transport dynamics through electrostatic or hydrogen‐bonding interactions.

Design strategies for intrinsic ion channels to enhance conductivity included: (a) Ligand functionalization: Grafting ‐SO_3_H or ‐PO_3_H_2_ acid groups to enhance proton conduction and incorporating crown ether or nitrogen‐rich heterocyclic ligands for selective chelation of alkali metal ions; (b) Metal node engineering: Utilizing open metal sites as low‐energy hopping sites for ion migration; (c) Pore environment tuning: Precisely adjusting pore size and geometry to match target ion dimensions and solvation shells; (d) Transport modes: Solvated ions diffuse along ligand‐arranged pathways or through pore cavities, often exhibiting low activation energy and thus low ionic transport resistance.

##### 6.2.2.2 Fabrication of Novel Architecture of 2D MOF to Strengthen the Electrochemical Stability and Mechanical Flexibility of SSEs

The pursuit of high‐performance SSEs has identified 2D MOFs as a uniquely advantageous material platform. Unlike their 3D counterparts or other inorganic solid electrolytes, 2D MOFs possess an inherent set of structural and chemical properties that can be strategically leveraged to simultaneously overcome the twin challenges of poor electrochemical stability and insufficient mechanical flexibility. Their advantages stem from their nanoscale thickness, tunable surface chemistry, and anisotropic structure.

##### 6.2.2.3 Advantages of 2D MOFs in Enhancing Electrochemical Stability

Electrochemical stability is crucial for SSE to withstand the reduction potential of lithium metal anodes and the oxidation potential of high‐voltage cathodes, where 2D MOFs demonstrate significant advantages. First, ligand chemistry can modulate the framework's highest occupied molecular orbital (HOMO) and lowest unoccupied molecular orbital (LUMO) energy levels. Introducing electron‐withdrawing groups like ‐F or ‐CF_3_ in ligands lowers HOMO energy, enhancing oxidation stability for high‐voltage cathodes. Conversely, Aromatic ligands confer reduction resistance at the anode. Second, the dense, ordered 2D layered structure acts as a physical barrier, more effectively inhibiting lithium dendrite growth compared to amorphous polymer or granular ceramic composite electrolytes. Third, functional groups such as ‐COO^−^ and ‐SO_3_^−^ on the surface of 2D MOF nanosheets promote the formation of a stable solid electrolyte interphase (SEI) rich in Li_2_O and LiF. This interface exhibits excellent ionic conductivity and mechanical properties.

##### 6.2.2.4 Advantages of 2D MOFs in Enhancing Mechanical Flexibility

The brittleness of inorganic SSE materials poses a significant bottleneck for their application in flexible electronics, and 2D MOFs offer a fundamental solution through their mechanical anisotropy. On the one hand, 2D MOF nanosheets possess intrinsic nanoscale flexibility. Their nanoscale thickness enables elastic bending and twisting without fracture—a capability unattainable in bulk 3D crystals or coarse‐grained ceramic electrolytes. On the other hand, high‐aspect‐ratio 2D MOF nanosheets readily composite with polymer matrices like PEO and PVDF. They form highly efficient mechanical permeation networks at low loading levels, simultaneously retaining the modulus and dendrite‐puncture resistance of the composite while maintaining the macroscopic flexibility of the matrix. Vacuum filtration can assemble them into self‐supporting “paper‐like” membranes, where interlayer forces enable flexible bending, and energy dissipation during nanoplate sliding confers toughness. Synergistic optimization of ion transport and mechanical properties can also be achieved through 3D scaffold architectures.

#### 6.2.3 Constructing Gradient Transition Layers

The integrated material electrolytes often introduces interfacial stress due to mismatches in physical properties, such as the coefficient of thermal expansion and elastic modulus. These stresses can compromise interfacial adhesion and overall structural stability. To mitigate this, constructing a functional gradient transition layer has proven effective [175, 176, 177]. Such a layer, composed of multiple strata of polymers or inorganic compounds with varying compositions, allows for a continuous gradient in properties like thermal expansion coefficient and modulus. Starting from the structural characteristics of 2D MOFs, modification strategies at the microscopic level can more precisely optimize interfacial contact. We can modify the structure by inserting ionic liquids (ILs) between layers [25]. Following insertion, ILs expand the interlayer spacing, providing broader transport pathways for Li^+^ and alleviating ion transport bottlenecks caused by interlayer stacking. Concurrently, cations within ILs form π‐π interactions with ligands within the 2D MOF layers, while anions establish weak coordination bonds with metal nodes. This approach stabilizes the interlayer structure against collapse while facilitating rapid Li^+^ migration at the interface through IL assistance. Furthermore, ILs form a dense interfacial layer on electrode surfaces to suppress side reactions. Their flexible nature also buffers interfacial stresses caused by electrode volume changes, ensuring long‐term contact stability. By carefully controlling the thickness and composition of each sub‐layer of 2D MOFs, this design effectively dissipates stress concentrations that arise from thermal cycling or mechanical deformation. Consequently, it significantly enhances the interfacial stability between 2D MOFs and adjacent materials, thereby improving the device's operational reliability and longevity.

#### 6.2.4 Development of Self‐Healing Surface Coatings

In the field of solid‐state electrolytes, a promising future application of 2D MOFs lies in the design of self‐repairing surface coatings to mitigate electrochemical‐mechanical failures induced by interfacial defects [178, 179, 180]. By incorporating dynamic chemical bonds that synergize with the vertical channel structure of 2D MOFs, composite systems exhibiting both high ionic conductivity and autonomous self‐healing capability can be constructed. Furthermore, the high aspect ratio and tunable active sites of 2D MOFs contribute to improved interfacial adhesion with electrodes, while the dynamic bilayer effect facilitates interfacial ion rearrangement and suppresses lithium dendrite growth. Future efforts may focus on optimizing the surface functionalization of 2D MOFs—integrating photo‐thermal or pH‐triggered self‐healing mechanisms—to enhance healing efficiency and environmental adaptability. Concurrently, scaling up the synthesis process will be essential to advancing practical implementation.

#### 6.2.5 Roll‐to‐Roll Preparation Processes

The roll‐to‐roll (R2R) process is a continuous and scalable manufacturing technology well‐suited for producing flexible materials [181, 182, 183]. For two‐dimensional 2D MOFs, the adoption of R2R techniques can enable their large‐scale fabrication in applications such as flexible electronics and energy storage devices. This approach involves the continuous processing of substrate materials through sequential steps—including deposition, coating, and reaction—to produce uniform 2D MOF films. The R2R process not only enhances production efficiency and reduces manufacturing costs, but also ensures high consistency in product quality, thereby facilitating the commercial deployment of 2D MOF‐based devices.

#### 6.2.6 Exploring All‐Solid‐State Thin‐Film Battery Applications

All‐solid‐state thin‐film batteries, with their high energy density, intrinsic safety, and long cycle life, are considered ideal energy storage solutions for next‐generation miniaturized and integrated electronics. The unique structure and properties of 2D MOFs offer significant potential in this field [184, 185, 186]. First, the 2D layered structure and atomic thickness of 2D MOFs enable the direct fabrication of large‐area, ultrathin, and dense continuous films via methods such as interfacial synthesis, vapor deposition, or electrochemical deposition. These films exhibit good substrate compatibility, and their thickness can be precisely controlled down to the nanoscale, greatly reducing ion transport distance and enhancing rate performance. Second, the highly ordered in‐plane channels and tunable interlayer spacing of 2D MOFs create unique “highways” for ion transport. Li^+^ can migrate rapidly through both the intra‐layer channels and the inter‐layer nanochannels, enabling a multi‐path conduction mechanism that may achieve ionic conductivity comparable to or exceeding that of traditional amorphous solid electrolytes. Moreover, 2D MOFs combine rigidity and flexibility. The rigid intralayer framework acts as a physical barrier against lithium dendrite penetration, while the interlayer flexibility and large surface area accommodate volume changes in lithium metal anodes, ensuring stable solid‐solid contact, reduced interfacial resistance, and extended cycle life. Additionally, 2D MOF films can be composited with polymers or inorganic electrolytes to form composite thin‐film electrolytes that simultaneously offer high ionic conductivity, mechanical strength, and a wide electrochemical window. This strategy helps bridge the physical and chemical mismatch between electrodes and electrolytes, which is critical for all‐solid‐state thin‐film batteries using high‐voltage cathodes and lithium metal anodes. This direction not only extends the application of 2D MOFs in energy storage but also offers a promising material‐based solution in all‐solid‐state batteries.

#### 6.2.7 Artificial Intelligence‐Assisted Screening

The growing diversity and compositional complexity of 2D MOFs pose significant challenges to conventional experimental screening methods, which are often time‐consuming and costly. The advancement of artificial intelligence (AI) offers a promising solution to this issue [187, 188]. By establishing extensive databases containing structural and property information of 2D MOFs and applying machine learning algorithms to analyze and model these data, AI enables accurate prediction of material performance. Furthermore, it facilitates the rapid identification of 2D MOFs with promising properties, thereby providing targeted guidance for subsequent experimental studies and accelerating the development of high‐performance materials.

## 7 Conclusion

2D MOFs demonstrate tremendous potential and have achieved significant research progress in the field of high‐performance solid‐state electrolytes. Their unique layered structures, characterized by ordered ion channels and tunable interlayer spaces, provide efficient 2D pathways for rapid ion transport. Simultaneously, favorable interlayer interactions confer excellent mechanical properties and structural stability. When employed as functional fillers in composite solid electrolytes, the ultra‐thin 2D structure and abundant open pores of 2D MOFs greatly enhance the contact interface with polymer matrices, optimize ion transport paths, and significantly boost ionic conductivity. Their inherent thermal and chemical stability improves overall electrolyte safety. The ease of structural regulation, functional group modification, and the ability to form synergistic composites with other materials make 2D MOFs an ideal platform for designing advanced solid‐state electrolytes.

Although challenges such as scalable preparation and interfacial side reactions persist, future developments in gradient self‐healing interfaces and roll‐to‐roll deposition processes are poised to achieve leapfrog improvements in material performance. Meanwhile, 2D MOFs also offer unique value for batteries beyond lithium‐ion. They can accommodate the larger ionic radii of Na^+^/K^+^ ions by adjusting interlayer spacing. Their surface active sites can reduce the migration energy barriers for Mg^2+^/Zn^2+^ ions. Their partially metal‐sulfur bonded frameworks can also withstand aqueous Zn^2+^ or high‐pressure Mg^2+^ electrolyte environments. Through precise channel tuning and functionalization, 2D MOFs hold promise as a universal electrolyte platform across energy storage systems. 2D MOF‐based solid‐state electrolytes hold great promise for building energy cores for long‐lasting wearable devices, high‐security fast‐charging electric vehicle batteries, and lifelong‐powered micro‐medical implants. Their continued development will be instrumental in driving the next generation of high‐performance, high‐safety energy storage systems and their deep integration into smart living.

## Conflicts of Interest

The authors declare no conflicts of interest.

## Data Availability

The authors have nothing to report.
